# 14-3-3 proteins inactivate DAPK2 by promoting its dimerization and protecting key regulatory phosphosites

**DOI:** 10.1038/s42003-021-02518-y

**Published:** 2021-08-19

**Authors:** Matej Horvath, Olivia Petrvalska, Petr Herman, Veronika Obsilova, Tomas Obsil

**Affiliations:** 1 Department of Physical and Macromolecular Chemistry, Faculty of Science, Charles University, Prague, Czech Republic; 2 Department of Structural Biology of Signaling Proteins, Division BIOCEV, Institute of Physiology of the Czech Academy of Sciences, Vestec, Czech Republic; 3 Institute of Physics, Faculty of Mathematics and Physics, Charles University, Prague, Czech Republic

**Keywords:** SAXS, X-ray crystallography, Kinases, Phosphoproteins

## Abstract

Death-associated protein kinase 2 (DAPK2) is a CaM-regulated Ser/Thr protein kinase, involved in apoptosis, autophagy, granulocyte differentiation and motility regulation, whose activity is controlled by autoinhibition, autophosphorylation, dimerization and interaction with scaffolding proteins 14-3-3. However, the structural basis of 14-3-3-mediated DAPK2 regulation remains unclear. Here, we structurally and biochemically characterize the full-length human DAPK2:14-3-3 complex by combining several biophysical techniques. The results from our X-ray crystallographic analysis revealed that Thr369 phosphorylation at the DAPK2 C terminus creates a high-affinity canonical mode III 14-3-3-binding motif, further enhanced by the diterpene glycoside Fusicoccin A. Moreover, concentration-dependent DAPK2 dimerization is disrupted by Ca^2+^/CaM binding and stabilized by 14-3-3 binding in solution, thereby protecting the DAPK2 inhibitory autophosphorylation site Ser318 against dephosphorylation and preventing Ca^2+^/CaM binding. Overall, our findings provide mechanistic insights into 14-3-3-mediated DAPK2 inhibition and highlight the potential of the DAPK2:14-3-3 complex as a target for anti‐inflammatory therapies.

## Abstract (web summary)

Horvath et al. structurally and biochemically characterize the full-length human DAPK2-14-3-3 complex to investigate the effects of binding to DAPK2 on its dimerization, activation by dephosphorylation of Ser318, and Ca^2+^/calmodulin binding. Their results provide mechanistic insights into 14- 3-3-mediated DAPK2 inhibition and highlight the potential of the DAPK2:14-3-3 complex as a target for anti-inflammatory therapies.

## Introduction

The death-associated protein kinase (DAPK) family consists of Ser/Thr protein kinases that control various cellular processes, including membrane blebbing, apoptosis, and autophagy^1–3^. In mammals, the DAPK family includes five proteins (DAPK1, DAPK2, DAPK3, DRAK1 and DRAK2) differing in their subcellular localization and binding partners despite containing catalytic domains with high sequence homology^4–7^. As the smallest member of the DAPK family, DAPK2 (also known as DRP-1) is involved in apoptosis, autophagy, granulocyte differentiation, and motility regulation. As such, DAPK2 is a potential target for anti‐inflammatory therapies^5,8–11^.

**Fig. 1:**
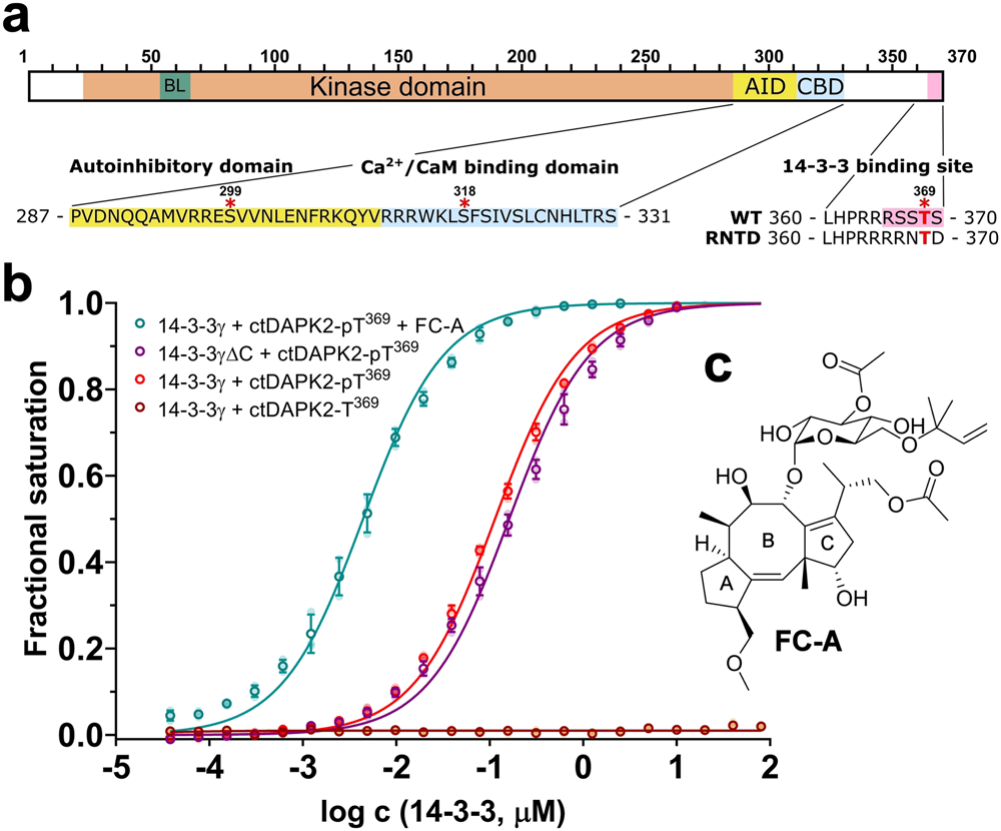
The C terminus of DAPK2 binds to 14-3-3 via a mode III binding motif. **a** Domain structure of human DAPK2. BL basic loop, AID autoinhibitory domain, CBD Ca^2+^/CaM-binding domain. The positions of the (auto)phosphorylation sites Ser^299^, Ser^318^, and Thr^369^, and the sequence of the DAPK2_RNTD_ mutant C terminus are indicated below. **b** Fluorescence polarization (FP) measurements of FAM-labeled ctDAPK-pT^369^ peptide with and without 100 μM Fusicoccin A (FC-A) titrated by 14-3-3γ (or 14-3-3γΔC). The binding affinities were determined by fitting the FP data to a one-site-binding model, with 161 ± 7, 114 ± 3, and 4.4 ± 0.2 nM apparent *K*_D_ for ctDAPK2-pT^369^ peptide binding to 14-3-3γΔC, 14-3-3γ, and 14-3-3γ + FC-A, respectively. All data points are the mean ± SD of three replicates. **c** FC-A structure.

DAPK2 is regulated in a Ca^2+^/calmodulin (Ca^2+^/CaM)-dependent manner and consists of an N-terminal kinase domain (KD, residues 23–285), which shares 80% homology with the kinase domain of DAPK1, followed by an autoinhibitory domain (AID, residues 287–311), a Ca^2+^/CaM-binding domain (CBD, residues 312–330) and a C-terminal tail with unique properties, thus lacking all other C-terminal domains of DAPK1 involved in protein–protein interactions (Fig. 1a)^4,5,12^. Several crystal structures of DAPK2 have been solved, covering the kinase and autoinhibitory domains of the protein, albeit without the 50-amino acid-long, presumably unstructured C-terminal tail^13,14^. Based on these crystal structures of DAPK2, the kinase domain is in an active conformation when occupied by the autoinhibitory region, which allows ATP, but not substrate binding^13,14^. Such an assembly suggests similar regulatory mechanisms shared between kinases activated by Ca^2+^/CaM-binding; accordingly, additional regulatory mechanisms, e.g., post-translational modifications (PTM) and/or interactions with other binding partners, may also be at play^15–17^.

DAPK2 regulation starts at the transcriptional level, where the methylation status of the DAPK2 promoter and spatiotemporal recruitment of specific transcription factors regulate DAPK2 expression^5,18–21^. Moreover, DAPK2 promoter hypermethylation is commonly found as a pro-survival marker in many cancers^22^. In turn, at the protein level, DAPK2 activity is regulated by autophosphorylation, which affects both inter- and intramolecular interactions of DAPK2. When recombinantly expressed in eukaryotic cells, DAPK2 is autophosphorylated at Ser^318^ within the CBD^4,5,23^. The phosphate group at this Ser^318^ has several inhibitory effects on the catalytic properties of DAPK2. For example, in the model proposed by Shani et al.^23^, phosphorylated Ser^318^ (pSer^318^) disrupts DAPK2 homodimerization and occupies the active site through interaction with Lys^151^. In addition, pSer^318^ adds a local negative charge near the Ca^2+^/CaM-binding site, thereby preventing the negatively charged Ca^2+^/CaM complex from binding to DAPK2^4,23^. Furthermore, the crystal structure of the DAPK2 homodimer has shown that its dimerization interface covers most of the kinase domain and part of the CBD, indicating that homodimerization is likely responsible for DAPK2 inhibition^13,14^. Therefore, this alternative model presents the inactive DAPK2 as a tightly packed homodimer in which both protomers are phosphorylated at Ser^318^, their active sites are blocked by AID, and their dimerization is mediated via KDs, including interactions between the basic loop in the kinase N-lobe, a unique feature of DAPKs, and the AID of the opposing protomer^14^.

DAPK2 activation presumably begins with the dissociation of its homodimer, followed by Ca^2+^/CaM binding to the low-affinity binding site located at the basic loop of DAPK2 (residues 55–65). This interaction triggers a small conformational change, exposing pSer^318^ to phosphatases. pSer^318^ dephosphorylation enables Ca^2+^/CaM to translocate to the CBD (residues 312–330) and to initiate another conformational change whereby the AID region is pulled from the kinase domain, thus exposing the active site for substrate binding^13,14,23^. Alternatively, the inhibitory effect of Ser^318^ phosphorylation can be bypassed through phosphorylation at Ser^299^, which activates DAPK2 in a Ca^2+^/CaM-independent/non-canonical manner. Because Ser^299^ is located at the hinge (residues 299–302) connecting KD to AID, the additional negative charge likely triggers a conformational change, which removes the AID from the KD and exposes pSer^318^ to phosphatases^11,24^. Although KD and AID are important for DAPK2 regulation, the mechanistic functions of other structural elements have been mostly overlooked in previous studies.

The flexible C-terminal tail of DAPK2, for example, may also play a key role in DAPK2 dimerization and in the regulation of its kinase activity based on available data^5,23,25^. Moreover, the C terminus of DAPK2 contains a motif recognized by scaffolding proteins 14-3-3, whose binding decreases DAPK2 activity, both in vitro and in cellulo, suggesting that 14-3-3 binding may suppress DAPK2 activity, in addition to autoinhibition and homodimerization^25,26^. Notwithstanding these functional findings, the exact role of 14-3-3 in DAPK2 regulation and the structural specificities of this interaction, in particular, have not been elucidated yet. In this context, to further our structural understanding of 14-3-3-mediated DAPK2 regulation, we prepared and characterized, structurally and biochemically, both the 14-3-3-binding motif of DAPK2 and full-length human DAPK2 in complex with the 14-3-3γ protein.

## Results

### DAPK2 possesses a high-affinity 14-3-3-binding motif further enhanced by Fusicoccin A

Previous studies have identified three canonical pSer/pThr-containing binding motifs recognized by 14-3-3 proteins: RXX(pS/T)XP (mode I), RXXX(pS/T)XP (mode II), and X(pS/T)X_1-2_COOH (mode III), where X denotes any amino acid^27–30^. Yuasa et al.^26^ showed that 14-3-3 binding to DAPK2 is regulated by Thr^369^ phosphorylation at the C terminus of DAPK2, which creates a mode III 14-3-3-binding motif RRRSSpT^369^S-COOH, where pT denotes phosphothreonine. Moreover, DAPK2 has been shown to interact with all human 14-3-3 proteins, and the 14-3-3γ protein often exhibits a higher binding affinity to various interaction motifs than other isoforms^26,31–33^. For this reason, we used 14-3-3γ throughout this work. To characterize this pThr^369^-dependent interaction between the C terminus of DAPK2 and 14-3-3γ, we performed fluorescence polarization measurements using synthetic C-terminal DAPK2 peptides with either Thr or pThr at position 369 and labeled at the N terminus by 5-carboxyfluorescein (denoted as FAM-ctDAPK2-T^369^ and FAM-ctDAPK2-pT^369^, respectively, sequences FAM-RRRSSTS and FAM-RRRSSpTS). Our measurements confirmed high-affinity binding to 14-3-3γ, with a *K*_D_ of 114 ± 3 nM for the phosphorylated peptide (Fig. 1b). As expected, no binding to the unphosphorylated peptide was detected. These results corroborate previously reported data, thus confirming that 14-3-3 binding to the C terminus of DAPK2 is mediated by Thr^369^ phosphorylation.

**Fig. 2:**
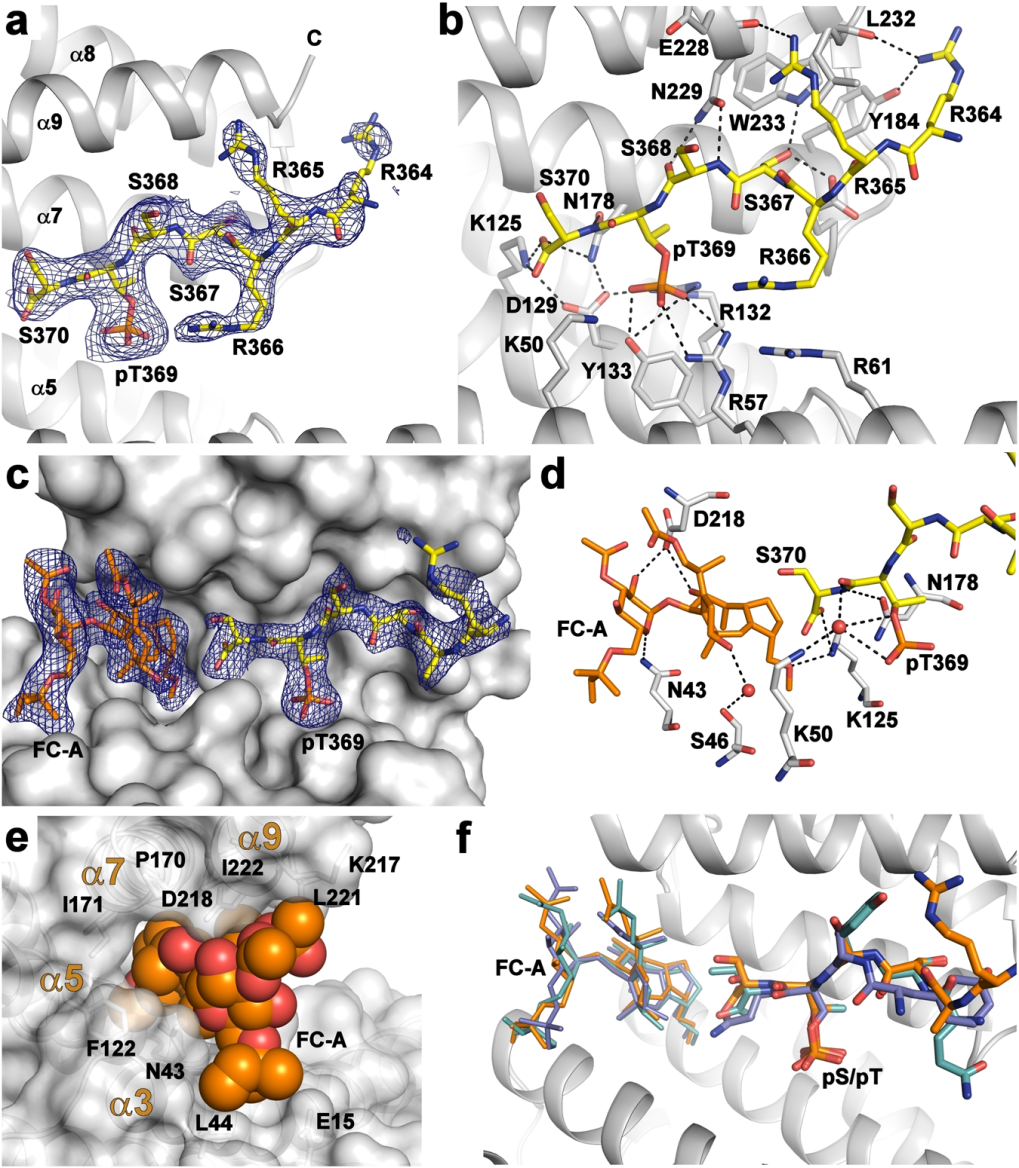
Structural analysis of interactions between the 14-3-3-binding motif pThr^369^ of DAPK2 and 14-3-3γ. **a** ctDAPK2-pT^369^ peptide (shown as sticks) bound within the binding groove of 14-3-3γ (shown as ribbon). The final 2*F*_O_ − *F*_C_ electron density map is contoured at 0.8σ. **b** Detailed view of contacts between 14-3-3γ and the ctDAPK2-pT^369^ peptide. The DAPK2 residues are shown in yellow, and 14-3-3γ residues in gray. Polar contacts are indicated by black dotted lines. **c** FC-A (shown as orange sticks) and the ctDAPK2-pT^369^ peptide (shown as yellow sticks) bound within the binding groove of 14-3-3γ (shown as gray surface). The final 2*F*_O_ − *F*_C_ electron density map is contoured at 1σ. **d** Detailed view of polar contacts between the ctDAPK2-pT^369^ peptide (yellow sticks) and FC-A (orange sticks). The 14-3-3γ residues are shown as gray sticks. **e** Detailed view of interactions between FC-A (orange spheres) and the 14-3-3 ligand-binding groove (gray surface). **f** Superimposition of the 14-3-3γ:ctDAPK2-pT^369^:FC-A (orange, this study), 14-3-3:C terminus of the plant plasma membrane H(+)-ATPase:FC-A (cyan, PDB ID: 1O9F) and 14-3-3:C terminus of plant KAT1 channel:FC-A (blue, PDB ID: 5NWK) ternary complexes. Only 14-3-3γ from the 14-3-3γ:ctDAPK2-pT^369^:FC-A complex is shown for clarity (gray ribbon).

To characterize the interaction between 14-3-3 and the putative 14-3-3-binding motif of DAPK2 at atomic resolution, we solved the crystal structure of the phosphopeptide ctDAPK2-pT^369^ (sequence RRRSSpT^369^S) bound to 14-3-3γΔC (ΔC denotes C-terminally truncated 14-3-3γ without the flexible ~13-residue-long C-terminal tail). Our FP measurements confirmed that 14-3-3γΔC binds to FAM-ctDAPK2-pT^369^ with a binding affinity similar to that of 14-3-3γ (*K*_D_ = 161 ± 7 nM, Fig. 1b). The 14-3-3γΔC:ctDAPK2-pT^369^ complex crystallized in the trigonal space group R3, with four 14-3-3:peptide molecules in the asymmetric unit. The structure was refined to a resolution of 2.7 Å (Table 1), and the final electron density allowed us to build all seven residues of the ctDAPK2-pT^369^ peptide. ctDAPK2-pT^369^ occupies both ligand binding grooves of the 14-3-3γ dimer in an extended conformation and interacting with 14-3-3γ as described in previously reported complexes with other mode III motifs (Fig. 2a, b)^28,34,35^. The phosphate moiety of pThr^369^ is coordinated by three 14-3-3γ residues, Arg^57^, Arg^132^ and Tyr^133^. The C-terminal Ser^370^ residue of DAPK2 forms two hydrogen bonds with the 14-3-3γ residues Lys^125^ and Asn^178^. Moreover, the ctDAPK2-pT^369^ makes several additional polar and hydrophobic contacts with residues from the 14-3-3 ligand-binding groove, including Glu^185^, Asn^229^, Leu^232^, and Trp^233^.

**Table 1:** Data collection and refinement statistics.

	14-3-3γ:ctDAPK2-pT^369^ (PDB ID: 7A6R)	14-3-3γ:ctDAPK2-pT^369^:FC-A (PDB ID: 7A6Y)
*Data collection*		
Space group	R3	R3
Cell dimensions		
*a*, *b*, *c* (Å)	205.62, 205.62, 74.09	205.50, 205.50, 74.27
*α*, *β*, *γ* (°)	90.0, 90.0, 120.0	90.0, 90.0, 120.0
Resolution (Å)	29.68–2.70 (2.80–2.70)^a^	29.68–2.50 (2.59–2.50)
*R*_meas_	9.6% (53.9%)	4.1% (29.2%)
*I*/σ*I*	13.5 (2.4)	26.4 (3.6)
Completeness (%)	99.7 (99.0)	99.6 (99.7)
Redundancy	4.39 (2.88)	4.23 (2.29)
*Refinement*		
Resolution (Å)	29.68–2.70 (2.80–2.70)	29.68–2.50 (2.59–2.50)
No. of reflections	31,974 (3165)	40,342 (4043)
*R*_work_/*R*_free_	0.2367/0.2754	0.2230/0.2590
No. of atoms		
Protein	7375	7284
Ligand/ion	0	144
Water	34	151
*B*-factors		
Protein	49.03	52.30
Ligand/ion	–	58.87
Water	41.26	47.22
R.m.s. deviations		
Bond lengths (Å)	0.002	0.002
Bond angles (°)	0.41	0.50

^a^Values in parentheses are for the highest-resolution shell.

Fusicoccin A (FC-A, Fig. 1c) is a diterpene glycoside phytotoxin known to stabilize 14-3-3 protein complexes^28,34,36–38^. Because DAPK2 has a mode III C-terminal motif, we hypothesized that FC-A may also stabilize the interaction between 14-3-3γ and ctDAPK2-pT^369^. Our FP experiments revealed that 100 µM FC-A increased the apparent binding affinity of FAM-ctDAPK2-pT^369^ ~26-fold to a *K*_D_ of 4.4 ± 0.2 nM (Fig. 1b). Furthermore, stabilization of the 14-3-3γ:ctDAPK2-pT^369^ complex by FC-A was also confirmed by differential scanning fluorimetry, which revealed a substantial increase in the apparent melting temperature (*T*_m_) of the 14-3-3γ:ctDAPK2-pT^369^ complex in the presence of FC-A (Supplementary Fig. S1).

To understand the interaction between 14-3-3γ, ctDAPK2-pT^369^ and FC-A, we solved the crystal structure of a ternary complex at 2.5 Å resolution (Table 1). The position and interactions of the ctDAPK2-pT^369^ peptide within the ligand-binding groove are similar to those in a binary complex (Supplementary Fig. S2). The diterpene moiety of FC-A is embedded within the 14-3-3-binding groove, between α-helices α3, α5, α7, and α9, forming several polar and hydrophobic interactions with 14-3-3γ residues (Fig. 2c, e). The glycosidic moiety of FC-A is more exposed to the solvent than the diterpene moiety and directly interacts with two 14-3-3γ residues, Asn^43^ and Asp^218^. The *O*-methyl group at ring A of FC-A (Fig. 1c) is hydrogen-bonded to the 14-3-3γ residue Lys^125^, which also interacts with Ser^370^ of ctDAPK2-pT^369^. No direct contacts between FC-A and ctDAPK2-pT^369^ were observed (Fig. 2d). In comparison with other FC-A complexes, in the 14-3-3γΔC:ctDAPK2-pT^369^:FC-A complex, FC-A makes similar contacts with the 14-3-3 ligand-binding groove but adopts a slightly different conformation (Fig. 2f, shown in orange), most likely due to the serine residue at the C terminus of the phosphopeptide. The other two FC-A complexes superimposed with the C termini of the H^+^ pump and the KAT1 channel (shown in Fig. 2f in cyan and blue, respectively) contain valine and asparagine, respectively, as their C-terminal residues^28,39^.

### The autophosphorylated full-length DAPK2 mutant DAPK2_RNTD_ shows similar properties to those of WT DAPK2

*E. coli* routinely used for recombinant protein expression lacks most of the PTM modifiers, thus affecting the activity or stability of recombinantly expressed proteins. Autophosphorylation of Ser^318^ located in the CBD is a crucial PTM that controls DAPK2 activity^4,5,23^. For this reason, we characterized the phosphorylation status of recombinantly expressed full-length DAPK2 by mass spectrometry, focusing on peptides containing regulatory phosphorylation sites Ser^318^ and Thr^369^ (Fig. 1a). Our LC–MS analysis confirmed substantial autophosphorylation of both DAPK2 phospho-sites (Supplementary Figs. S3a and S4a). These peptides contained either one or two phosphoresidues, and their unphosphorylated forms were not detected. 14-3-3 binding requires only singly phosphorylated motifs because the presence of two phosphoresidues in one 14-3-3-binding motif reduces 14-3-3 binding^40^. Similarly, Yuasa et al.^26^ observed that the DAPK2 mutants S367A, S368A, and S370A form a more stable complex with 14-3-3 than DAPK2 WT, thus suggesting that the presence of other phosphoresidue(s), in addition to pThr^369^, at the C terminus of DAPK2 inhibits complex formation. To avoid this negative effect while maintaining the integrity of the mode III 14-3-3-binding motif, we designed a DAPK2 mutant fully competent in binding to 14-3-3 (Fig. 1a, denoted as DAPK2_RNTD_), whose C terminus –RSST^369^S-COOH was replaced by the sequence –RRNT^369^D-COOH. This sequence resembles a well-characterized 14-3-3-binding motif III from the C terminus of serotonin *N*-acetyltransferase (–RRNSDR-COOH)^29^ with only one phosphorylatable Thr residue at the same position of this residue in DAPK2 WT. Our LC–MS analysis revealed that recombinantly expressed DAPK2_RNTD_ is stoichiometrically autophosphorylated at the C-terminal Thr^369^ (Supplementary Fig. S4b), thus requiring no additional phosphorylation to create a functional 14-3-3-binding motif. This autophosphorylation of the DAPK2_RNTD_ peptide containing Ser^318^ was similar to that of DAPK2 WT (Supplementary Fig. S3b). The phosphorylation status of peptides selected for analysis was confirmed by MS/MS. An example of such MS/MS spectra is shown in Supplementary Fig. S5. Furthermore, the specific kinase activity of DAPK2_RNTD_ (31.3 ± 0.2 nmol min^−1^ mg^−1^, measured using the peptide containing the N-terminal part of the Myosin regulatory light chain, sequence KKRAARATSNVFA, as the substrate) was also similar to that of DAPK2 WT (30.9 ± 0.3 nmol min^−1^ mg^−1^) (Supplementary Fig. S6a). In line with previous reports, the kinase activity of DAPK2_RNTD_ decreased significantly in the presence of 14-3-3 proteins (Supplementary Fig. S6b)^25,26^. Hence, we used DAPK2_RNTD_ for the biophysical and structural characterization of interactions between DAPK2 and 14-3-3γ.

### Ca^2+^/CaM-binding blocks concentration-dependent DAPK2 dimerization

**Fig. 3:**
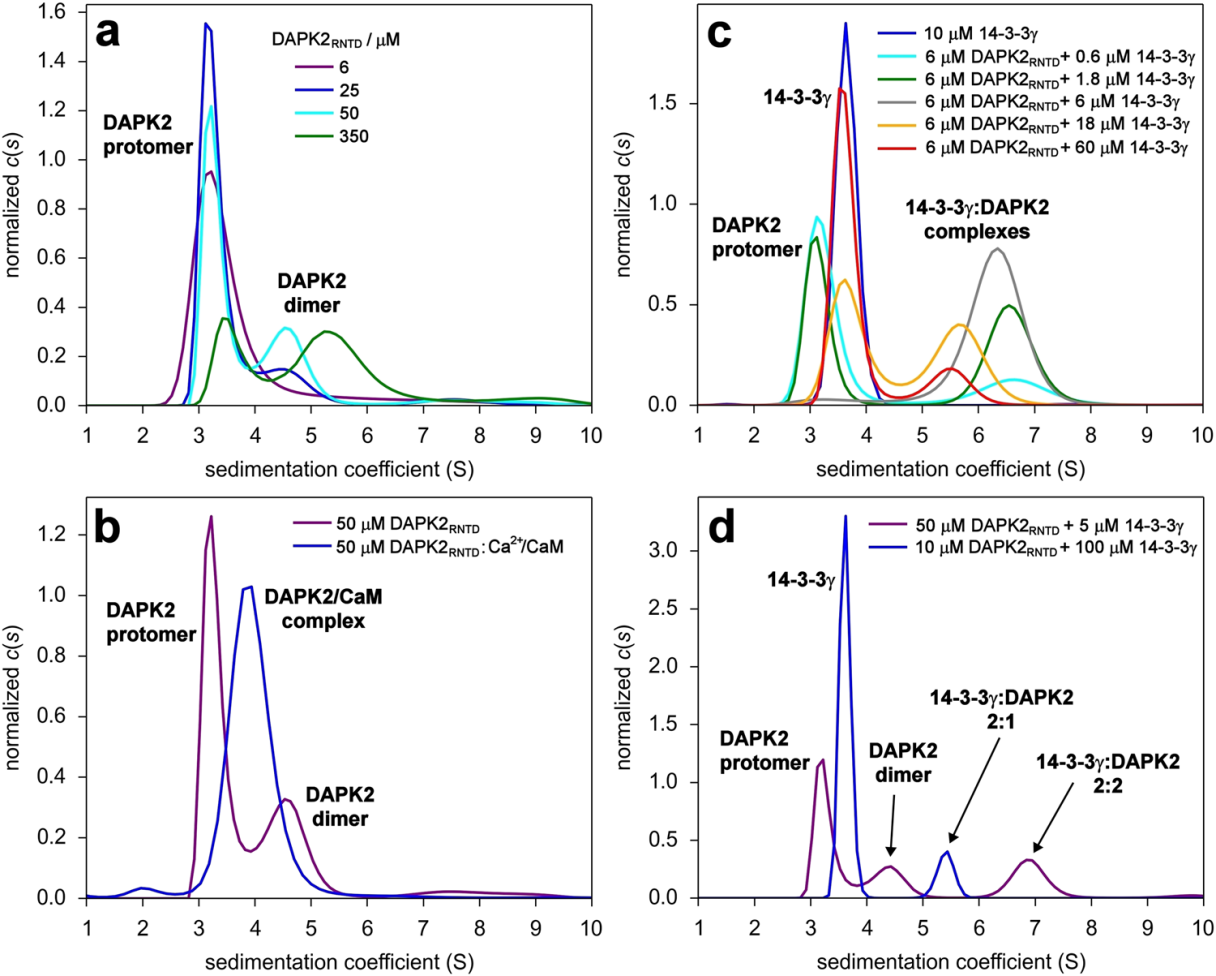
Sedimentation velocity analytical ultracentrifugation analysis of the DAPK2_RNTD_ and its interactions with Ca^2+^/CaM and 14-3-3γ. **a** Series of area-normalized *c*(*s*) distributions of DAPK2_RNTD_ alone at various concentrations. **b** Comparison of area-normalized *c*(*s*) distributions of 50 μM DAPK2_RNTD_ alone and of a 50-μM mixture of DAPK2_RNTD_ with Ca^2+^/CaM (1:1 molar ratio). **c** Series of area-normalized *c*(*s*) distributions of mixtures of DAPK2_RNTD_ and 14-3-3γ at various molar ratios, using 6 μM DAPK2_RNTD_ and 0.6–60 μM 14-3-3γ. **d** Comparison of area-normalized *c*(*s*) distributions of mixtures of DAPK2_RNTD_ with 14-3-3γ at 10:1 (violet) or 1:10 (blue) molar ratios.

DAPK2_RNTD_ oligomerization in solution was characterized by sedimentation velocity analytical ultracentrifugation (SV-AUC). At a low protein concentration (6 μM), the sedimentation coefficient distribution *c*(*s*) exhibited only one peak, with a weight-averaged sedimentation coefficient (corrected to 20.0 °C and to the density of water), *s*_w(20,w)_, of 3.6 S (estimated *M*_w_ ∼40 kDa). In contrast, at high protein concentrations (25−350 μM), the *c*(*s*) distributions showed an additional peak at high *s* values, most likely corresponding to the DAPK2_RNTD_ dimer (Fig. 3a). This peak suggested a concentration-dependent self-association of DAPK2_RNTD_, with a *K*_D_ of ∼100 μM (based on the ratios of the peak areas), thus corroborating previous reports^13,14^. Ca^2+^/CaM binding blocked this homodimerization, as indicated by the *c*(*s*) distribution of the equimolar mixture of DAPK2_RNTD_ and Ca^2+^/CaM (Fig. 3b), which contained only one peak, with a *s*_w(20,w)_ of 4.1 S, corresponding to a *M*_w_ ∼ 60 kDa (the theoretical *M*_w_ of the DAPK2_RNTD_:Ca^2+^/CaM complex is 60.2 kDa). In addition, the formation of a stable complex with Ca^2+^/CaM also implies that DAPK2 phosphorylated at Ser^318^ can still interact with Ca^2+^/CaM.

### DAPK2 and 14-3-3γ form complexes with two different stoichiometries

We investigated the interaction between DAPK2_RNTD_ and 14-3-3γ by SV-AUC analysis of their mixtures at various molar ratios (6 μM DAPK2_RNTD_ and 0.6–60 μM 14-3-3γ) (Fig. 3c). The shift in the peak corresponding to the complex whose *s* values decrease with the increase in the concentration of 14-3-3γ, especially in samples with a high excess of 14-3-3γ (orange and red distributions in Fig. 3c), suggested the formation of complexes with different stoichiometries. This was confirmed by analyzing samples with a 10-molar excess of either DAPK2_RNTD_ or 14-3-3γ over the other (Fig. 3d). When 14-3-3γ was in excess, a peak with a *s*_w(20,w)_ value of 5.7 S (estimated *M*_w_ ∼ 106 kDa) appeared, most likely representing the DAPK2_RNTD_:14-3-3γ complex with a 2:1 stoichiometry (theoretical *M*_w_ 100.2 kDa). Conversely, when DAPK2_RNTD_ was in excess, a peak with a *s*_w(20,w)_ value of 7.2 S (estimated *M*_w_ ∼ 149 kDa) was observed, most likely representing the DAPK2_RNTD_:14-3-3γ complex with a 2:2 stoichiometry (and a theoretical *M*_w_ of 143.4 kDa). We were unable to determine the apparent *K*_D_ of the 14-3-3γ:DAPK2_RNTD_ interaction from the titration experiment because DAPK2_RNTD_ and 14-3-3γ formed complexes with different stoichiometries. Nevertheless, the *c*(*s*) distribution of the sample containing 6 μM 14-3-3γ and 6 μM DAPK2_RNTD_ (Fig. 3c, gray trace) indicated a minimal presence of free 14-3-3γ and DAPK2_RNTD_, thus suggesting a *K*_D_ value lower than 100 nM, assuming a 2:2 stoichiometry for the complex.

### 14-3-3γ promotes DAPK2 dimerization and interacts with its AID and CBD

To gain structural insights into 14-3-3-mediated DAPK2 regulation, we tried to crystallize the DAPK2_RNTD_:14-3-3γ complex. Despite our extensive screening, all crystallization trials were unsuccessful, presumably due to the dynamic nature of this complex. Nevertheless, small-angle X-ray scattering (SAXS) and chemical cross-linking coupled to mass spectrometry (MS) are frequently used to study flexible and conformationally heterogeneous systems. Furthermore, these methods have been previously applied to the structural characterization of other 14-3-3 complexes^41–45^. Therefore, we used this alternative approach based on SAXS and cross-linking MS to characterize our DAPK2_RNTD_:14-3-3γ complex.

**Fig. 4:**
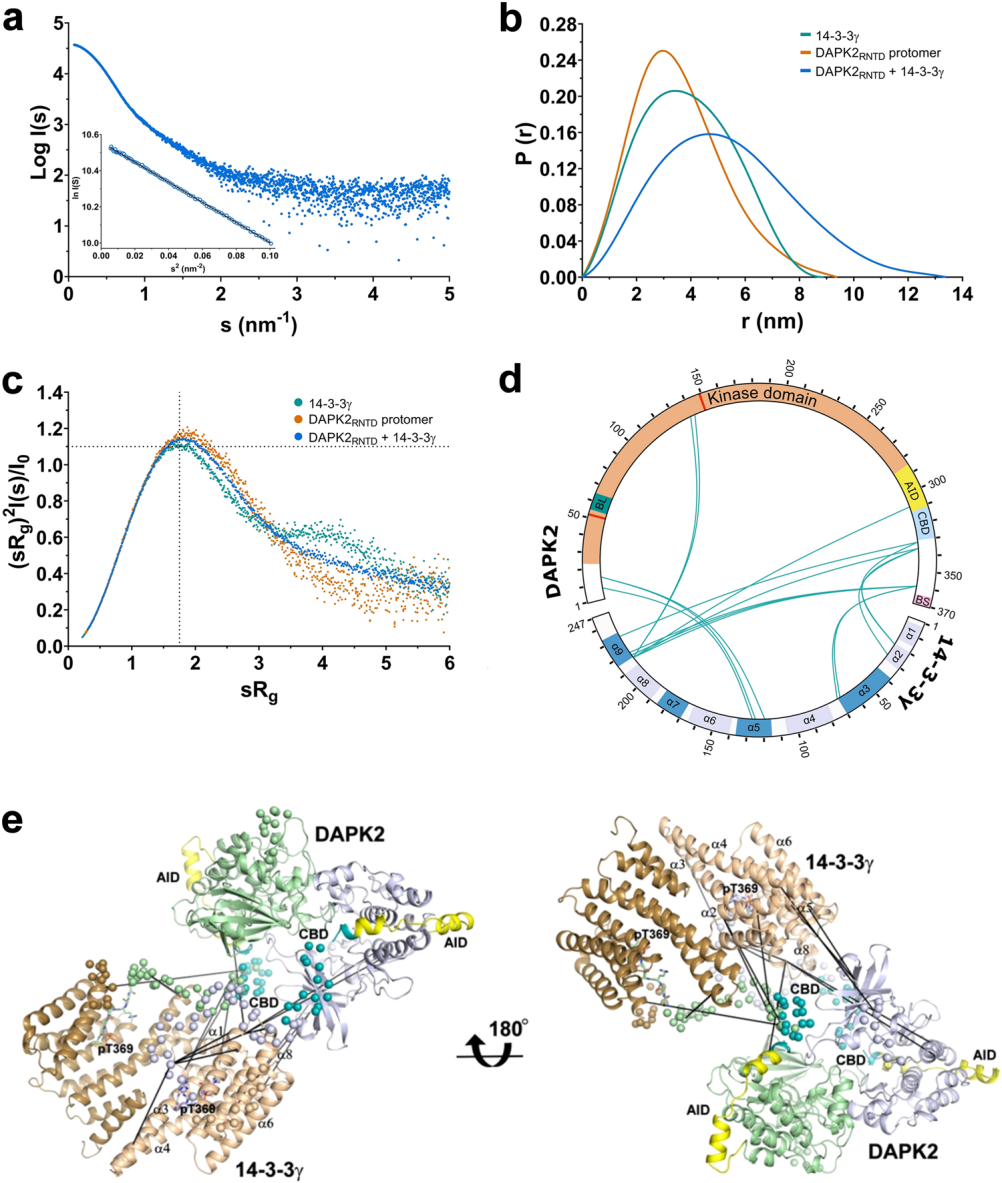
SAXS-based structural characterization of the DAPK2_RNTD_:14-3-3γ complex. **a** Scattering intensity as a function of the scattering vector *s* (*s* = 4πsin(*θ*/*λ*), where 2*θ* is the scattering angle, and *λ* is the wavelength) of the DAPK2_RNTD_:14-3-3γ complex. The inset shows the Guinier plot of the scattering data. **b** Distance distribution functions *P*(*r*) calculated from scattering data. **c** Dimensionless Kratky plots. Dotted lines mark the maximum at a value of 1.104 for *sR*_g_ = 1.73, which is typical of scattering data of compact globular proteins^46^. **d** Unique cross-links between DAPK2 and 14-3-3γ mixed in a 1:1 molar ratio with a 50-fold molar excess of the cross-linking agent DSG are represented by green lines. DAPK2 diagram: Kinase domain (orange), Basic loop (BL, green), Autoinhibitory domain (AID, yellow), Calmodulin-binding domain (CBD, light blue), 14-3-3-binding site (BS, pink). Red lines within the kinase domain represent key residues in ATP binding. 14-3-3 diagram: colored regions represent α-helices of the 14-3-3 molecule, whereas those that form the ligand-binding groove are colored in dark blue. This figure was prepared using xiVIEW (https://xiview.org/) and InkScape (http://www.inkscape.org/). **e** The best-scoring model of the DAPK2_RNTD_:14-3-3γ complex was constructed using the program CORAL^47^ and the crystal structures of autoinhibited DAPK2 (PDB ID: 2A2A^14^) and the 14-3-3γ:ctDAPK2-pT^369^ complex (PDB ID: 7A6R, this study). The unstructured segments missing in the crystal structures were modeled as dummy residue chains (shown as spheres). The AID and CBD of DAPK2 are colored in yellow and cyan, respectively. The ctDAPK2-pT^369^ peptide is shown as sticks. Black lines connect C_α_ atoms of DAPK2 to 14-3-3γ residues cross-linked by DSG.

We characterized the structure of the DAPK2_RNTD_:14-3-3γ complex in solution by analyzing both DAPK2_RNTD_ alone and the 14-3-3γ and DAPK2_RNTD_ mixture at a 1:3 molar ratio by size exclusion chromatography (SEC) coupled to SAXS (Supplementary Fig. S7a). The scattering data of 14-3-3γ were collected in batch mode (Supplementary Fig. S7d). The X-ray scattering profile of the DAPK2_RNTD_:14-3-3γ complex and the calculated *P*(*r*) distance distribution function (Fig. 4a, b) revealed a molecular weight (*M*_w_), a radius of gyration (*R*_g_), and a maximal distance within the particle (*D*_max_) of 147 kDa, 40.9 Å, and 133 Å, respectively (Supplementary Table S1). The estimated *M*_w_ value matched the theoretical *M*_w_ of the DAPK2_RNTD_:14-3-3γ complex with a 2:2 stoichiometry (143.4 kDa). SEC-SAXS analysis of DAPK2_RNTD_ alone confirmed its partial dimerization, as shown by frames from the right side of the elution peak indicating particles with an estimated *M*_w_ of 44 kDa (the theoretical *M*_w_ of the DAPK2_RNTD_ protomer is 43.2 kDa). In contrast, frames from the left side of the elution peak indicate particles with an estimated *M*_w_ of 56 kDa, most likely corresponding to a mixture of DAPK2_RNTD_ protomers and dimers (Supplementary Fig. S7a–c). The DAPK2_RNTD_ protomer exhibited a *D*_max_ similar to that of 14-3-3γ, albeit considerably smaller than the *D*_max_ of the complex (Fig. 4b). The dimensionless Kratky plot ((*sR*_g_)^2^*I*(*s*)/*I*_0_ versus *sR*_g_) of the DAPK2_RNTD_:14-3-3γ complex (Fig. 4c) showed a bell-shaped curve with a maximum at *sR*_g_ ∼ 1.81, thus suggesting the increased conformational flexibility of the complex because of the scattering data for compact globular proteins (such as 14-3-3γ) in this plot peak at 1.104 at *sR*_g_ ~ 1.73^46^. In summary, DAPK2_RNTD_ partly dimerizes when alone in solution and forms a complex with 14-3-3γ with a 2:2 stoichiometry when in excess.

SAXS-based rigid-body structural modeling of the complex was performed using CORAL^47^ and the crystal structures of autoinhibited DAPK2 (PDB ID: 2A2A^14^) and the 14-3-3γ:ctDAPK2-pT^369^ complex (PDB ID: 7A6R, this study). The starting conformation of the DAPK2:14-3-3γ complex was first modeled as two isolated DAPK2 protomers bound to the 14-3-3γ dimer through their C-terminal pT^369^ motifs (Supplementary Fig. S8a). These simulations suggested that both DAPK2 protomers are located close to one another within the complex (Supplementary Fig. S8b) and that the DAPK2_RNTD_:14-3-3γ complex with the 2:2 stoichiometry consists of the DAPK2 dimer bound to the 14-3-3γ dimer. For this reason, we subsequently used a different starting conformation with the DAPK2 dimer^14^ bound to the 14-3-3γ dimer via pThr^369^-containing motifs of both DAPK2 protomers. The best-scoring model from these simulations fitted the experimental SAXS data with *χ*^2^ = 1.10 (Supplementary Fig. S8c) and positioned the DAPK2 dimer outside the central channel, close to the α8-α9 loop of 14-3-3γ. In this model, the flexible C-terminal segments of the DAPK2 protomers are located within the central channel of the 14-3-3γ dimer and at the interface of both proteins (Fig. 4e). Moreover, the CORAL model of the DAPK2 dimer:14-3-3γ dimer complex is consistent with the ab initio shape reconstruction calculated from the scattering data using DAMMIF (Supplementary Fig. S8d).

Chemical cross-linking of the DAPK2_RNTD_:14-3-3γ complex (mixed in a 1:1 molar ratio) by disuccinimidyl glutarate (DSG) enabled us to further characterize interactions between DAPK2 and 14-3-3γ (Supplementary Table S2 and Supplementary Fig. S9). Most intermolecular cross-links (cross-links #6−16) connect the C-terminal segment of DAPK2 containing AID, CBD, and the 14-3-3-binding motif to 14-3-3γ residues of the helices that form either the ligand-binding groove (helices α3 and α9) or the surface of the 14-3-3γ dimer central channel (helix α2) (Fig. 4d, e), in line with our SAXS-based model. Cross-links #1−3 connect the N terminus of DAPK2 to the 14-3-3γ helix α5 and cross-links #4,5 link the N terminus of 14-3-3γ helix α9 to the C-lobe of DAPK2 KD, thus indicating the existence of multiple conformations of the complex, which differ in the orientation of the dimer of DAPK2 kinase domains with respect to 14-3-3γ.

Combined, our SAXS and chemical-cross-linking data suggested that the DAPK2_RNTD_:14-3-3γ complex with a 2:2 stoichiometry consists of a DAPK2 dimer bound to a 14-3-3γ dimer and that 14-3-3γ interacts not only with the pT^369^-containing motif but also with AID and CBD, key regulatory DAPK2 regions.

### 14-3-3γ binding to DAPK2 destabilizes the interaction between DAPK2 and Ca^2+^/CaM

**Fig. 5:**
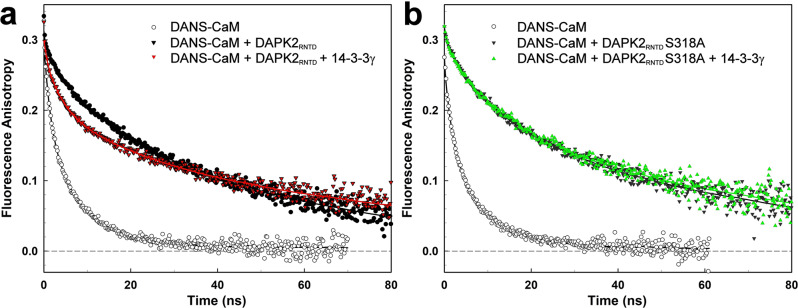
Time-resolved dansyl fluorescence measurements. **a** Fluorescence anisotropy decays of free dansyl-Ca^2+^/CaM (open circles) and dansyl-Ca^2+^/CaM in the presence of DAPK2_RNTD_ (black circles), and DAPK2_RNTD_ and 14-3-3γ (red triangles). **b** Fluorescence anisotropy decays of free dansyl-Ca^2+^/CaM (open circles) and dansyl-Ca^2+^/CaM in the presence of DAPK2_RNTD_S318A (black triangles), and DAPK2_RNTD_S318A and 14-3-3γ (green triangles).

SAXS-based modeling and chemical cross-linking suggested that 14-3-3γ interacts with the CBD-containing region of DAPK2 (Fig. 4d, e). Thus, we assessed whether DAPK2_RNTD_ association with 14-3-3γ interferes with the Ca^2+^/CaM binding to this protein kinase. For this purpose, we prepared dansyl-labeled CaM (DANS-CaM) and monitored its binding to DAPK2_RNTD_ based on time-resolved fluorescence intensity and anisotropy decay measurements (Fig. 5 and Supplementary Table S3). As shown in Fig. 5a, the substantially slower fluorescence anisotropy decay, resulting from the increased value of the longest correlation time (compare *ϕ*_3_ = 8.1 ns for free Ca^2+^/CaM and *ϕ*_4_ = 52 ns in the presence of DAPK2_RNTD,_ Supplementary Table S3), clearly indicates the formation of the complex, with the consequent decrease in the overall rotational diffusion coefficient of DANS-Ca^2+^/CaM (Fig. 5a). Furthermore, DANS-Ca^2+^/CaM binding to DAPK2_RNTD_ also induced a significant increase in the mean excited-state lifetime (*τ*_mean_) of the dansyl moiety of ~4.6 ns, i.e., from 15.9 to 20.5 ns. This increase in the *τ*_mean_ of the dansyl moiety suggests its shielding from the polar environment upon formation of the DANS-Ca^2+^/CaM:DAPK2_RNTD_ complex.

14-3-3γ addition to the DAPK2_RNTD_:Ca^2+^/CaM complex considerably affected the dansyl fluorescence anisotropy decay in the region of shorter correlation times, as shown by the raw data (red triangles in Fig. 5a), indicating increased dansyl mobility caused by 14-3-3γ. A more rigorous data analysis revealed a new subnanosecond component with the correlation time *ϕ*_1_ and a rise in the *β*_2_ amplitude, with an approximately 3.6-ns correlation time. We detected a similar correlation time, with comparable amplitude, in DANS-CaM with and without 14-3-3γ (see the first two rows of Supplementary Table S3) and a substantial decrease in the amplitude *β*_4_ associated with the longest rotational correlation time *ϕ*_4_, which reflects the amount of DANS-Ca^2+^/CaM:DAPK2_RNTD_ complex. These changes and the decrease in *τ*_mean_ likely resulted from the increased exposure of the dansyl moiety to the polar environment, suggesting that 14-3-3γ binding to DAPK2_RNTD_ partly dissociates the DANS-Ca^2+^/CaM:DAPK2_RNTD_ complex. The presence of 14-3-3γ alone had no effect on DANS-CaM fluorescence (Supplementary Fig. S10a).

The same experiments were performed with the DAPK2_RNTD_S318A mutant as well, albeit with no changes either in dansyl anisotropy decay or in *τ*_mean_ in the presence of 14-3-3γ (green triangles in Fig. 5b). The residue Ser^318^ is located within the CBD of DAPK2 (Fig. 1a), and its phosphorylation has been suggested to prevent Ca^2+^/CaM binding to DAPK2^23,48^. However, DAPK2_RNTD_ and DAPK2 WT, both containing autophosphorylated Ser^318^ (Supplementary Fig. S3), were able to interact with Ca^2+^/CaM, as indicated by our SV-AUC and dansyl fluorescence measurements (Figs. 3b, 5a and Supplementary Fig. S10b). Nevertheless, the ability of 14-3-3γ to dissociate Ca^2+^/CaM from DAPK2_RNTD_ but not from DAPK2_RNTD_S318A suggests that pSer^318^ indeed destabilizes the interaction between DAPK2 and Ca^2+^/CaM, thus enabling CaM dissociation upon 14-3-3γ binding to DAPK2.

### 14-3-3γ binding protects Ser^318^ and Thr^369^ of DAPK2 from dephosphorylation

As previously suggested, one of the possible phosphatases responsible for DAPK2 dephosphorylation and subsequent activation is calcineurin, whose overexpression considerably suppressed the binding of DAPK2 to 14-3-3γ^26^. 14-3-3 proteins regulate the function of their binding partners by protecting regulatory phosphorylation sites against dephosphorylation, among other mechanisms^44,49,50^. Thus, we subsequently assessed whether 14-3-3γ binding slows down the dephosphorylation of DAPK2 regulatory phosphosites Ser^318^ and Thr^369^. Calcineurin is a protein phosphatase activated similarly to DAPK2 by Ca^2+^/CaM binding; hence, we used the Mn^2+^-dependent type I protein phosphatase (PP1) to avoid any potential interference of Ca^2+^/CaM during dephosphorylation. The results of the dephosphorylation reaction were analyzed by Mn^2+^ Phos-tag SDS-PAGE. This phosphate-affinity electrophoresis technique separates phosphorylated from non-phosphorylated proteins. When comparing the relative abundances of various DAPK2_RNTD_ phospho-forms and their time-dependent downward shift (Supplementary Fig. S11), we noted a gradual change in DAPK2_RNTD_ phospho-fingerprint, observing the first changes after 5 min of reaction, and most DAPK2_RNTD_ was dephosphorylated after a 240-min-long incubation. Overnight exposure to PP1 resulted in almost complete dephosphorylation of DAPK2. In the presence of 14-3-3γ, the phospho-fingerprints also changed, albeit at a considerably slower rate. Most phospho-sites were protected for at least 30 min, detecting phosphorylated residues even after overnight exposure to PP1.

**Fig. 6:**
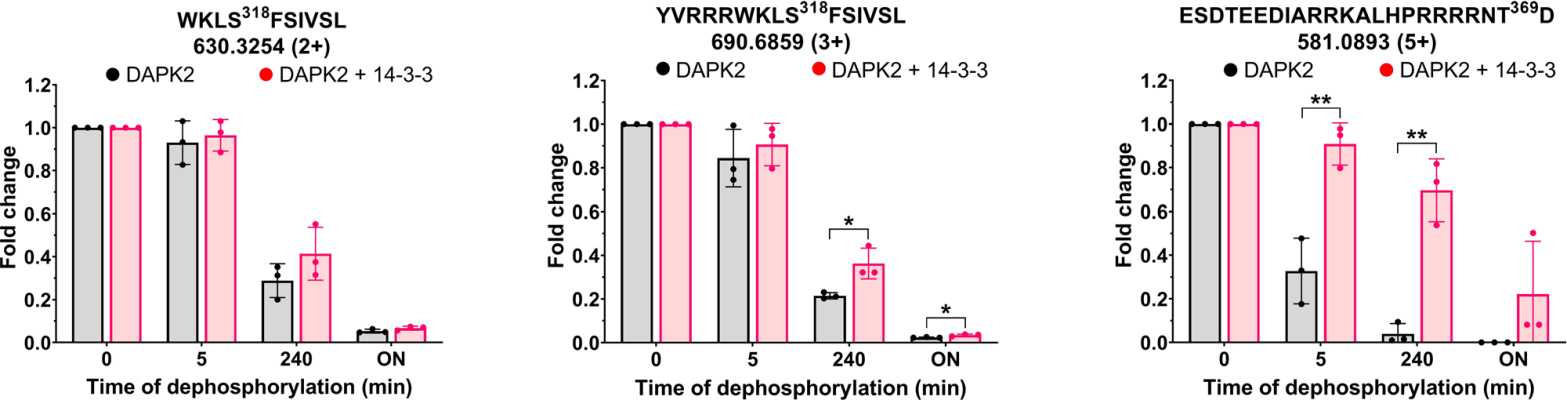
14-3-3γ slows down DAPK2 dephosphorylation at Ser^318^ and Thr^369^. Relative abundances of phosphorylated DAPK2_RNTD_ peptides containing pSer^318^ and pThr^369^ after 5 m, 2 h and overnight dephosphorylation of DAPK2_RNTD_ by PP1 with and without 14-3-3γ. Phosphopeptides were quantified by LC–MS. Error bars represent the standard deviation of three independent experiments. Asterisks represent significant differences according to Student’s *t*-tests comparing changes between selected time points (^n.s.^*P* > 0.05; *P ≤ 0.05; **P ≤ 0.01).

By LC–MS, we then characterized the phosphorylation status of the regulatory phosphorylation sites Ser^318^ and Thr^369^ within the C-terminal segment of DAPK2 exposed to PP1, with and without 14-3-3γ. We estimated the abundances of selected phosphorylated peptides based on their intensities extracted from ion chromatograms and normalized using a factor calculated from the intensities of three non-phosphorylated peptides. We followed modifications in Ser^318^ using two different peptides, W^315^KLSFSIVSL^324^ and Y^310^VRRRWKLSFSIVSL^324^ (peptides were selected based on S/N ratio), albeit detecting both single and doubly phosphorylated forms of these peptides. Accordingly, the additional serine residue(s) that are present in these peptides (Ser^320^ and Ser^323^) can also be autophosphorylated (Supplementary Fig. S3). However, the proximity of all three serine residues in these peptides prevented us from accurately identifying the second modified phosphoserine. We assessed the phosphorylation status of Thr^369^ using the C-terminal peptide E^348^SDTEEDIARRKALHPRRRRNTD^370^, detecting only a singly phosphorylated form of this peptide. The comparison of DAPK2_RNTD_ phosphopeptide abundances with and without 14-3-3γ revealed that 14-3-3γ binding protected both sites against dephosphorylation by PP1 and that Thr^369^ of the 14-3-3-binding motif was considerably better protected than Ser^318^ (Fig. 6).

## Discussion

In solution, 14-3-3 binding to DAPK2 protects the DAPK2 inhibitory autophosphorylation site Ser^318^ against dephosphorylation, thereby preventing Ca^2+^/CaM binding to DAPK2 and consequently its activation. Furthermore, 14-3-3γ protein promotes DAPK2 dimerization, which also maintains this protein kinase in its inactive state. Although previous research had suggested that DAPK2 phosphorylation at the C-terminal Thr^369^ triggers 14-3-3 binding, decreasing DAPK2 activity both in vitro and in cellulo^25,26^, the exact role of 14-3-3 in DAPK2 regulation, especially its structural specificities, had not been elucidated until now. Thus, our study provides key biochemical and structural insights into the mechanism of 14-3-3-mediated DAPK2 inhibition by combining several analytical methods.

Previous research has revealed that four residues within the C-terminal 14-3-3-binding motif of human DAPK2 (residues S^367^STS^370^) are phosphorylated in vivo^51^. Accordingly, our LC–MS analysis of human recombinant DAPK2 WT showed that DAPK2 is autophosphorylated not only at regulatory Ser^318^ (Supplementary Fig. S3a) but also at two additional sites within the C-terminal 14-3-3-binding motif, at least (Supplementary Fig. S4a). However, Yuasa et al.^26^ demonstrated that the interaction of 14-3-3 proteins with DAPK2 depends mainly on the phosphorylation of Thr^369^ because only the DAPK2 mutant T369A exhibits a significantly lower DAPK2 binding to 14-3-3ε than DAPK2 WT, whereas the DAPK2 mutants S367A, S368A, and S370A formed a more stable complex with 14-3-3ε. Indeed, our crystallographic analysis and fluorescence polarization measurements revealed that Thr^369^ phosphorylation at the position −1 from the C terminus creates a high-affinity canonical mode III 14-3-3-binding motif whereby 14-3-3γ binds to DAPK2, as in other mode III motifs (Fig. 2)^28,34,35^. This binding can be further strengthened by FC-A (Figs. 1b and 2c), which targets a gap in the interface between the 14-3-3 ligand-binding groove and some 14-3-3-binding motifs, especially those of type III^28,34,36–38^. Nevertheless, DAPK2 binding to 14-3-3 proteins may also be mediated by phosphorylation of one of the serine residues preceding Thr^369^ because previous studies have shown that the DAPK2 T369A mutant retains some ability to interact with 14-3-3 proteins^25,26^.

SV-AUC analysis of DAPK2_RNTD_ stoichiometrically autophosphorylated at Ser^318^ and Thr^369^ (Supplementary Figs. S3b and S4b), which is fully competent in 14-3-3 binding, confirmed that DAPK2 undergoes concentration-dependent dimerization (Fig. 3a). In turn, this dimerization can be blocked by Ca^2+^/CaM binding (Fig. 3b), indicating that either CBD is a part of the DAPK2 dimerization interface or Ca^2+^/CaM binding to the DAPK2 CBD disrupts the contacts responsible for DAPK2 KD dimerization. These findings corroborate the previous report by Simon et al.^14^, who observed a partial overlap between the CaM-binding site and the dimerization interface and who showed that the basic loop of the DAPK2 KD is also involved in Ca^2+^/CaM binding as a key structural element of the dimerization interface.

**Fig. 7:**
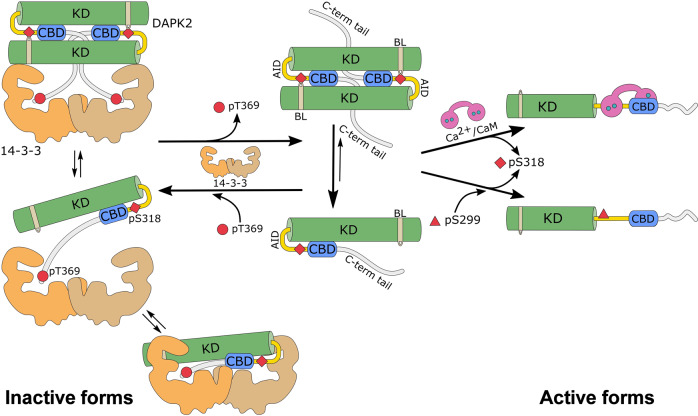
DAPK2 inhibition depends on both autophosphorylation and interaction with the 14-3-3 protein. In solution, DAPK2 is in equilibrium between the *cis*-autoinhibited monomer or *trans*-autoinhibited dimer. Under physiological conditions, the equilibrium is shifted towards the monomeric form. In both cases, DAPK2 is locked in the autoinhibited conformation mediated by Ser^318^ autophosphorylation (represented as a red diamond). The phosphorylation of the C-terminal Thr^369^ (red circle), either by autophosphorylation or by PKB^26^ (or some other still unidentified kinase), creates a mode III 14-3-3-binding site. Because the 14-3-3 dimer contains two binding grooves, 14-3-3 can increase the local DAPK2 concentration and shift the equilibrium towards the DAPK2 dimer. Accordingly, the inactive form of DAPK2 is a *trans*-autoinhibited dimer with autophosphorylated Ser^318^ and Thr^369^ in a complex with the 14-3-3 dimer, which stabilizes the dimeric form of DAPK2, protects Ser^318^ against dephosphorylation, and prevents Ca^2+^/CaM binding to DAPK2. Alternatively, 14-3-3 proteins interact with only one molecule of DAPK2, which could allow a bidentate interaction in which the 14-3-3 dimer simultaneously interacts with both the C-terminal motif and the pSer^318^-containing motif within AID. This bidentate interaction would also protect pSer^318^ against dephosphorylation and destabilize Ca^2+^/CaM binding to DAPK2. In short, both Ca^2+^/CaM-dependent and -independent (based on Ser^299^ phosphorylation^24^, represented as a red triangle) DAPK2 activation require Thr^369^ and Ser^318^ dephosphorylation.

14-3-3 proteins are well known to form stable dimers that anchor two binding partner molecules, especially when the binding partner contains only one 14-3-3-binding motif^52,53^. Unsurprisingly, our SV-AUC measurements with mixtures of DAPK2_RNTD_ and 14-3-3γ demonstrated the formation of complexes with not only 1:2 (DAPK2:14-3-3 dimer) but also 2:2 (Fig. 3d) stoichiometries in a molar excess of 14-3-3 and in DAPK2 concentrations higher than those of 14-3-3, respectively. On the one hand, because 14-3-3 proteins are highly expressed in various tissues^31,54,55^, the complex with a 1:2 stoichiometry is likely more abundant than the complex with a 2:2 stoichiometry. On the other hand, 14-3-3 proteins interact with several hundred binding partners^56,57^. Therefore, the availability of specific 14-3-3 proteins may be locally limited, which could promote the formation of DAPK2:14-3-3 complexes with a 2:2 stoichiometry. Thus, it is likely that the two types of complexes coexist (Fig. 7). We tried to prepare and structurally characterize the complex with a 1:2 stoichiometry, but we were unable to obtain X-ray scattering data for this complex, most likely due to the formation of complexes with two different stoichiometries. Nevertheless, we successfully extracted SAXS data for the DAPK2_RNTD_:14-3-3γ complex with a 2:2 stoichiometry from the left side of the elution peak for the DAPK2_RNTD_ and 14-3-3γ mixture at a 3:1 molar ratio (Fig. 4a and Supplementary Table S1, Supplementary Fig. S7a). Our SAXS-based modeling suggested that this complex consists of a DAPK2 dimer bound to the 14-3-3γ dimer rather than two DAPK2 protomers bound to the 14-3-3γ dimer (Fig. 4e and Supplementary Fig. S8a, b). Moreover, the complex with a 2:2 stoichiometry did form in 6 μM DAPK2_RNTD_, which does not dimerize at this concentration (Fig. 3a), further supporting our model according to which 14-3-3 binding promotes DAPK2 self-association. Because DAPK2 dimerization precludes substrate binding^13,14^, this 14-3-3-mediated stabilization of DAPK2 dimers may contribute to the inhibitory effect of 14-3-3 binding (Supplementary Fig. S5b)^25,26^.

The interaction between DAPK2 and 14-3-3γ in the complex with 1:2 stoichiometry may differ from that in the complex with a 2:2 stoichiometry. Thus, additional contacts between 14-3-3 and DAPK2 cannot be ruled out when complexes of both stoichiometries are formed. Furthermore, the sequence surrounding the autophosphorylation site Ser^318^ (R^312^RRWKLpSFSIV^322^) within CBD resembles 14-3-3-binding motifs (Fig. 1a). The FP measurements with the FAM-labeled peptide containing this sequence revealed weak binding to 14-3-3γ (Supplementary Fig. S12a). Therefore, we cannot exclude the possibility that the DAPK2_RNTD_:14-3-3γ complex with a 1:2 stoichiometry is formed through a bidentate interaction in which the 14-3-3γ dimer simultaneously interacts with both phosphorylated motifs of DAPK2 (Fig. 7 and Supplementary Fig. S12b), as previously shown in other 14-3-3-binding partners^52,58–60^.

Previously reported crystal structures of DAPK2 kinase domain dimers have also suggested that autophosphorylated Ser^318^ within CBD likely interacts with arginine residues in the basic loop of the opposing protomer^13,14^. Hence, 14-3-3-mediated stabilization of the DAPK2 dimer (or direct binding to the pSer^318^-containing motif in the complex with a 1:2 stoichiometry) may concomitantly protect this site against dephosphorylation, one of the initial steps of DAPK2 activation^23,48^. In our study, limited DAPK2 dephosphorylation by PP1 coupled to MS analysis showed that 14-3-3γ binding protected both Ser^318^ and Thr^369^ from dephosphorylation (Fig. 6c). In a similar mechanism, 14-3-3 keeps CaMKK kinases in their inhibited states by slowing down the dephosphorylation of the serine residue located within the Ca^2+^/CaM-binding region^44,49,50^. Considering this evidence, 14-3-3 also prevents Ser^318^ dephosphorylation, blocking DAPK2 activation.

In a third inhibitory mechanism, 14-3-3 establishes several contacts with the CBD of DAPK2 (Fig. 4d, e) and thus may also prevent Ca^2+^/CaM binding, based on chemical cross-linking and SAXS-based modeling. Concurrently, time-resolved fluorescence measurements with DANS-labeled CaM further supported our hypothesis that 14-3-3γ protein can dissociate Ca^2+^/CaM from DAPK2_RNTD_, albeit only when pSer^318^ is present because 14-3-3γ had no effect on Ca^2+^/CaM binding to the DAPK2_RNTD_S^318^A mutant (Fig. 5). These findings are in line with previous results demonstrating that pSer^318^ destabilizes the interaction between DAPK2 and Ca^2+^/CaM^23,48^. On balance, 14-3-3γ protein likely dissociates Ca^2+^/CaM from DAPK2 through a pSer^318^-dependent mechanism. However, the destabilization of the DAPK2_RNTD_:Ca^2+^/CaM complex observed in the presence of 14-3-3γ (Fig. 5a) was not complete, thus suggesting that Ca^2+^/CaM and 14-3-3 proteins may simultaneously bind to DAPK2, especially in the absence of Ser^318^ phosphorylation.

14-3-3 proteins regulate the function of several dozens of kinases, and more than 170 kinases contain putative 14-3-3-binding sites^56^. Yet, the mechanistic details of these processes of 14-3-3-mediated kinase regulation remain mostly elusive, except for the B-RAF kinase. The recently solved high-resolution structures of B-RAF:14-3-3 complexes have enabled us to understand in detail the role of 14-3-3 binding^52,60,61^. In this context, our data provide the first structural glimpse into 14-3-3-mediated DAPK2 regulation and suggest that 14-3-3 proteins regulate kinases through several common mechanisms, such as promoting or blocking their dimerization, protecting their key regulatory phosphosites, and interfering with their protein–protein interactions.

In conclusion, 14-3-3 binding inhibits DAPK2 through three interconnected mechanisms (Fig. 7). In solution, resting DAPK2 is in equilibrium between the cis-autoinhibited monomer and the *trans*-autoinhibited dimer. Under physiological conditions, the equilibrium is shifted towards the monomeric form. In both cases, DAPK2 is locked in the autoinhibited conformation mediated by Ser^318^ autophosphorylation, and C-terminal Thr^369^ phosphorylation, either by autophosphorylation or by PKB^26^ (or another, yet unidentified, kinase), creates a high-affinity mode III 14-3-3-binding motif. Because two binding motifs can simultaneously bind to the 14-3-3 dimer, 14-3-3 proteins increase the local concentration of DAPK2 and shift the equilibrium towards the DAPK2 dimer. Therefore, we propose that the physiologically relevant DAPK2 inactive form is a *trans*-autoinhibited dimer with phosphorylated Ser^318^ and Thr^369^ in a complex with the 14-3-3 protein dimer, which stabilizes DAPK2 dimers, thereby protecting Ser^318^ against dephosphorylation and preventing Ca^2+^/CaM binding and that Ca^2+^/CaM-dependent and -independent DAPK2 activation pathways require both Ser^318^ and Thr^369^ dephosphorylation. Alternatively, 14-3-3 proteins may interact with only one DAPK2 molecule, which could allow a bidentate interaction in which the 14-3-3 dimer simultaneously interacts with both the C-terminal motif and the pSer^318^-containing motif within AID. This bidentate interaction would also protect pSer^318^ against dephosphorylation and destabilize Ca^2+^/CaM binding to DAPK2.

Three questions, nevertheless, remain to be answered in subsequent studies, namely (i) whether the autophosphorylation at the C terminus is of any physiological relevance or merely an effect of the high DAPK2 concentration during its overexpression in bacterial cells, (ii) whether the stabilization of protein–protein interactions in DAPK2:14-3-3 complexes by small-molecule compounds may be a practical strategy for inhibiting DAPK2 activity and (iii) what is the predominant stoichiometry of DAPK2:14-3-3 complexes in vivo. Yet, the potential applications of our results are only limited by the scope of cellular processes in which DAPK2 and 14-3-3 proteins participate. Accordingly, our findings should trigger future research on apoptosis, autophagy, and tumor suppression, with a translational output, ranging from prediction studies to structure-based drug discovery.

## Materials and methods

### Recombinant protein expression and purification

Human 14-3-3γWT and 14-3-3γΔC (residues 1-235 lacking the C-terminal 13-residue-long flexible tail) were expressed and purified as previously described^62^. Purified 14-3-3γ proteins in a buffer containing 20 mM HEPES-NaOH (pH 7.5), 150 mM NaCl, 5 mM DTT and 10% (w/v) glycerol were flash-frozen in liquid nitrogen and stored in aliquots at −80 °C. In turn, recombinant DAPK2 proteins were expressed using a plasmid containing full-length human DAPK2 kindly provided by William Hahn & David Root (Addgene plasmid #23390; http://n2t.net/addgene:23390; RRID: Addgene_23390)^63^. For this purpose, DNA encoding *DAPK2* was ligated into the modified pRSFDuet-1 vector (Merck KGaA, Darmstadt, Germany) using the *BamH*I and *Not*I sites (Supplementary Table S4). Modified pRSFDuet-1 containing the sequence of the His_6_-tagged *GB1* domain of protein G inserted into the first multiple cloning site was a gift from Evzen Boura (Institute of Organic Chemistry and Biochemistry AS CR, Prague, Czech Republic). The *DAPK2*_*RNTD*_ mutant was prepared by mutating the last four amino acid residues S^367^STS^370^ to R^367^NTD^370^ (Supplementary Table S4) using the QuikChange site-directed mutagenesis kit (Stratagene, La Jolla, CA, USA). The *DAPK2*_*RNTD*_ S318A was prepared from DAPK2_RNTD_ by mutating Ser^318^ to alanine using the same procedure. All constructs were evaluated by sequencing from both termini. DAPK2 proteins were expressed in *Escherichia coli* Rosetta (DE3) cells (Merck KGaA, Darmstadt, Germany) grown in LB media supplemented with chloramphenicol, kanamycin, and 0.5 mM IPTG; for 18 h at 25 °C and 190 rpm. DAPK2 was purified by immobilized metal ion affinity chromatography using the Chelating Sepharose Fast Flow resin (GE Healthcare, Chicago, IL, USA) according to a standard protocol. The eluted protein was dialyzed overnight at 8 °C against a buffer containing 20 mM HEPES (pH 7.5), 150 mM NaCl, 5 mM EDTA, 5 mM β-mercaptoethanol, and 10% (w/v) glycerol. The His_6_-GB1 tag was removed by adding tobacco etch virus (TEV) protease (250 U TEV/mg recombinant protein) to the eluted protein prior to dialysis. To completely remove the tag, the dialyzed protein was incubated for another 2 h at room temperature. The final purification step was size exclusion chromatography on a HiLoad Superdex 75 PG 26/600 column (GE Healthcare, Chicago, IL, USA) in a buffer containing 20 mM HEPES (pH 7.5), 150 mM NaCl, 5 mM DTT, 10% (w/v) glycerol. Purified protein was flash-frozen in liquid nitrogen and stored in aliquots at −80 °C. Lastly, rat calmodulin protein (rat CaM) was prepared as described previously^64^.

### DAPK2 dephosphorylation

DAPK2 dephosphorylation was performed in 250 µL of a reaction mixture containing 6 nmol DAPK2_RNTD_ and 12 nmol 14-3-3γ in NEB Protein Metallo-Phosphatases buffer containing 50 mM HEPES-NaOH (pH 7.5), 100 mM NaCl, 2 mM DTT, 0.01% (v/v) Brij 35 supplemented with 1 mM MnCl_2_ and 6 units of PP1 phosphatase (New England Biolabs, Ipswich, MA, USA) at 21 °C. Samples were collected at specific time points of 5 min, 15 min, 30 min, 1 h, 2 h, 4 h, and overnight (18 h). The reaction was stopped by adding β-glycerol phosphate to a final concentration of 25 mM and immediately flash-frozen in liquid nitrogen or mixed with Laemmli buffer and boiled for 5 min at 95 °C. The frozen samples were analyzed by HPLC-MS, and the samples mixed with Laemmli buffer were analyzed by 100 μM Mn^2+^-Phos-tag™ 10% SDS-PAGE (FUJIFILM Wako Chemicals Europe GmbH, Neuss, Germany).

### Fluorescence polarization (FP)-binding assay

Fluorescence polarization measurements were performed using a CLARIOstar microplate reader (BMG Labtech, Germany) on 384-well, black, low-volume, flat-bottom plates (Corning, USA) with peptides FAM-ctDAPK2-pT^369^ (sequence FAM-RRRSSpTS), FAM-ctDAPK2-T^369^ (sequence FAM-RRRSSTS) and FAM-aidDAPK-pS^318^ (sequence FAM-RRRWKLpSFSIV) (Pepscan Presto BV, The Netherlands), in a buffer containing 20 mM HEPES-NaOH (pH 7.4), 150 mM NaCl, 0.1% (v/v) Tween 20 and 0.1% (w/v) BSA. Excitation and emission wavelengths were 482 and 530 nm, respectively. In addition, 160 µM 14-3-3γ protein and its binary dilution series were incubated for 1 h with 50 nM FAM-ctDAPK-pT^369^ peptide and 100 µM FC-A (where needed), before the fluorescence polarization measurements. To determine the *K*_D_ values, the resulting curves were fitted to a one-site-binding model using OriginPro 2018b (OriginLab Corp. MA, USA).

### Analytical ultracentrifugation

Sedimentation velocity (SV) experiments were performed using a ProteomLabTM XL-I analytical ultracentrifuge (Beckman Coulter, Brea, CA, USA), as previously described^65^. Samples were dialyzed against a buffer containing 50 mM HEPES-NaOH (pH 7.5), 150 mM NaCl, and 1 mM TCEP before the AUC measurements. SV experiments were conducted in charcoal-filled Epon centerpieces with 12-mm optical path length at 20 °C and at 42,000 rev./min rotor speed (An-50 Ti rotor, Beckman Coulter, Brea, CA, USA). All sedimentation profiles were collected by absorbance at 280 nm. The calculated distributions were integrated to establish the weight-average sedimentation coefficients corrected to 20 °C and to the density of water, *s*_w(20,w)_.

### Small-angle X-ray scattering

Synchrotron SAXS data were collected at beamline P12 operated by EMBL Hamburg at the PETRA III storage ring (DESY, Hamburg, Germany). All proteins were dialyzed overnight before the SAXS measurements in a buffer containing 50 mM HEPES-NaOH (pH 7.5), 150 mM NaCl, 1 mM TCEP, and 5% (w/v) glycerol. The scattering data of 14-3-3 γ (3.4 mg mL^−1^) were collected in batch mode. The scattering data of DAPK2_RNTD_ (6.6 mg mL^−1^) and DAPK2_RNTD_:14-3-3γ complex (11.3 mg mL^−1^, mixed in 3:1 molar ratio) were collected in in-line SEC-SAXS mode using a Superdex 200 Increase 5/150 GL column (GE Healthcare, Chicago, IL, USA) at a flow rate of 0.5 mL min^−1^. The forward scattering *I*(0) and the radius of gyration *R*_g_ were calculated using the Guinier approximation for the *s* (*s* = 4πsin(*θ*)/*λ*, where 2*θ* is the scattering angle, and *λ* is the wavelength) range, which satisfies the *sR*_g_ < 1.3 condition. SEC-SAXS data were processed using CHROMIXS^66^. The distance distribution functions *P*(r) and the maximum particle dimensions *D*_max_ were calculated using GNOM^67^. The excluded volume of the hydrated particle (the Porod volume, *V*_P_) was calculated using PRIMUS^68^. The program DAMMIF^69^ was used to calculate ab initio molecular envelopes. Multiple iterations of DAMMIF were averaged using DAMAVER^70^. The rigid-body modeling of the 14-3-3γ:DAPK2_RNTD_ complex was performed using CORAL^47^, which models disordered loops missing in crystal structures as interconnected dummy residue chains attached to the appropriate Cα atoms in rigid domains. Crystal structures of the 14-3-3γ:ctDAPK2-pT^369^ complex (PDB ID: 7A6R) and the kinase domain of DAPK2 (PDB ID: 2A2A) were used as rigid domains. The calculated molecular envelope was aligned to structural models using SUPCOMB^71^.

### Differential scanning fluorimetry (DSF)

The differential scanning fluorimetry experiments were performed using a real-time PCR LightCycler 480 II (Roche Applied Science, Penzberg, Germany), according to the standard protocol^72^. The thermal stability of 14-3-3γΔC at a concentration of 7.5 µM was tested in the presence of 200 µM ctDAPK2-pT^369^ and 500 µM FC-A (Sigma-Aldrich, St. Louis, MO, USA), in a total reaction volume of 25 µL in buffer containing 20 mM HEPES (pH 7.5) and 2 mM MgCl_2_.

### Crystallization, data collection, and structure determination

Recombinantly expressed and purified 14-3-3γΔC protein and the ctDAPK2-pT^369^ peptide, sequence RRRSSpTS, (Pepscan Presto BV, The Netherlands) were mixed in a 1:1.2 molar stoichiometry in a buffer containing 20 mM HEPES (pH 7), 2 mM MgCl_2_, and 1 mM TCEP. Crystallization was performed using the hanging-drop vapor-diffusion method at 290 K, and crystals of the 14-3-3γΔC:ctDAPK2-pT^369^ binary complex were grown from drops consisting of 3 µL of 15.9 mg mL^−1^ protein and 3 µL of 100 mM HEPES (pH 7.5); 200 mM MgCl_2_; 25% (w/v) PEG400; 1% (v/v) hexafluoro-2-propanol. The 14-3-3γΔC:ctDAPK2-pT^369^:FC-A ternary complex was prepared by soaking the crystals of the binary 14-3-3γΔC:ctDAPK2-pT^369^ complex with 0.5 mM FC-A for 1 h at 290 K. Crystals were flash-frozen in liquid nitrogen. Diffraction data sets were collected on a MicroMax-007 HF Microfocus X-ray generator at a wavelength of 1.54187 Å with a VariMax VHF Arc Sec optical system (Rigaku, Japan), an AFC11 partial four-axis goniometer (Rigaku, Japan), a PILATUS 300K detector (Dectris, Switzerland) and a Cryostream 800 cryocooling system (Oxford Cryosystems, England, 100 K temperature). Diffraction data were processed using the XDS package^73^. Crystal structures of binary and ternary complexes were solved by molecular replacement in MOLREP^74^, using the structure of 14-3-3γ (PDB ID: 2B05) as a search model, and refined at 2.7 and 2.5 Å resolution, respectively, using the PHENIX package^75^. The final models of the 14-3-3γ:ctDAPK2-pT^369^ and the 14-3-3γ:ctDAPK2-pT^369^:FC-A complexes contained 99.78% and 99.44%, respectively, of the residues within favored regions of the Ramachandran plot and 0% of outliers. All structural figures were prepared with PyMOL (https://pymol.org/2/).

### Characterization of the phosphorylation status of the autophosphorylated peptides by liquid chromatography–mass spectrometry (LC–MS)

All LC–MS measurements were performed on a HPLC 1200 series (Agilent Technologies) connected to a 15T-SolariX XR^TM^ Fourier Transform Ion-Cyclotron-Resonance Mass Spectrometer (FT-ICR-MS, Bruker Corp., Billerica, MA, USA). All proteins were digested online on a Nepenthesin-2 column (66 µL bed volume) in 0.4% Formic acid in water at 400 µL min^−1^ flow rate. The resulting peptides were trapped and desalted with the same buffer composition on a reversed-phase trap column (ACQUITY UPLC BEH C18, 130 Å, 1.7 µm, 2.1 mm × 5 mm, Waters, Milford, MA, USA). The desalted peptides were eluted and separated on an analytical reversed-phase column (ACQUITY UPLC BEH C18, 130 Å, 1.7 µm, 1 mm × 100 mm, Waters, Milford, MA, USA) with a 10–45% linear gradient of Solvent B (solvent A: 0.1% Formic acid in water, solvent B: 0.1% Formic acid, 2% water in Acetonitrile) at 40 µL min^−1^ flow rate. The column was connected directly to a FT-ICR-MS operated in positive data-dependent mode using collisional-induced dissociation. The raw data were processed in Data Analysis 5.0 (Bruker Corp., Billerica, MA, USA), and the peptides were identified using MASCOT (Matrix Science Ltd., UK) against a database containing DAPK2, 14-3-3γ, and Nepenthesin-2 (the only partial modifications allowed were Ser/Thr/Tyr phosphorylation, cysteine carbamidomethylation and single methionine oxidation).

### Chemical cross-linking coupled to mass spectrometry

DAPK2_RNTD_ and 14-3-3γ protein stocks were diluted and mixed in 1:1 stoichiometry to a final concentration of 30 µM. The cross-linking reaction was performed in a buffer containing 20 mM HEPES-NaOH (pH 7.0), 150 mM NaCl and 1 mM TCEP and started by adding a 50-fold molar excess of disuccinimidyl glutarate (DSG, 1:1 molar ratio mixture of DSG-H6 and DSG-D6) (Creative Molecules Inc., Victoria, Canada). The cross-linking reaction was performed at RT and stopped after 30 min by adding 4× NuPage LDS sample buffer (ThermoFisher Science, Waltham, MA, USA). Proteins were separated on NuPage 4–12% Bis-Tris Protein Gels (ThermoFisher Science, Waltham, MA, USA) using NuPage MES SDS Running Buffer (ThermoFisher Science, Waltham, MA, USA). The band corresponding to the DAPK2_RNTD_:14-3-3γ complex with a 2:2 stoichiometry was excised from the gel and distained. Cysteines were reduced in a buffer containing 100 mM DTT, 50 mM NH_4_HCO_3_ (pH 8.5) at 60 °C for 30 min. Subsequently, the free cysteines were alkylated using 20 mM iodoacetamide in 50 mM NH_4_HCO_3_ (pH 8.5) at RT for 20 min in complete darkness. In-gel trypsin digestion was performed at 37 °C and quenched by adding 0.1% Trifluoroacetic acid after overnight incubation. Samples were loaded on a trap column (ZORBAX 300SB-C18, 5 µm, 5 × 0.3 mm, Agilent, Santa Clara, CA, USA), desalted for 5 min at flow rate 20 µL min^−1^ and then separated by reversed phase C18 column (ZORBAX SB C18 RR, 3.5 µ, 150 × 0.3 mm, Agilent, Santa Clara, CA, USA) at a flow rate 10 µL min^−1^ using capillary HPLC system (Agilent Technologies) using the acetonitrile gradient: 1–10% B in 1 min, 10–45% B in 19 min, 45–95% B in 5 min, where solvent A was 0.1% formic acid, 2.0% acetonitrile in water and solvent B was 0.1% formic acid in 98% acetonitrile. The column was heated at 50 °C and connected directly to a FT-ICR-MS operated in positive data-dependent mode using collisional-induced dissociation. Data were processed by DataAnalysis 5.0 software (Bruker Daltonics) exported to mgf file. Modified peptides were identified using the StavroX v3.6.6.0 program (http://www.stavrox.com/). The StavroX algorithm was set to consider cysteine carbamidomethylation and single methionine oxidation. The mass error threshold was below 1 ppm, and all assigned peptides were verified manually.

### Enzyme-activity measurements

DAPK2 kinase activity was assessed using the ADP-Glo^TM^ kinase assay (Madison, WI, USA), according to the manufacturer’s instructions. To compare the kinase activity of DAPK2_RNTD_ with that of DAPK2 WT, 50 nM DAPK2 was incubated with 250 nM CaM, 2.5 µM 14-3-3 (where needed), 10 µM ATP and 100 μM MLC peptide for 30 min at RT (24 °C) in the kinase reaction buffer containing 50 mM HEPES-NaOH (pH 7.5), 0.5 mM CaCl_2_, 20 mM MgCl_2_, 0.1 mM DTT, 0.01% BSA (w/v). Luminescence was measured in Greiner LUMITRAC™ 200 384-well plates on an Infinite^®^ 200 Pro plate reader (Tecan, Switzerland) with 1-s acquisition and 5-ms settle time between well measurements. No filters or attenuation methods were used during the measurements.

### CaM dansyl labeling

CaM was dansyl-labeled as described previously^76^. Briefly, the required amount of CaM was dialyzed against 10 mM NaHCO_3_ (pH 10.0) and diluted to 1 mg mL^−1^. After drop-wise addition of dansyl chloride (Sigma-Aldrich, St. Louis, MO, USA) from a 6 mM acetone stock to a final concentration of 90 µM, the sample was incubated for 45 min at 30 °C and then for another 18 hours at 8 °C. The excess of dansyl chloride was removed by size exclusion chromatography using a HiLoad Superdex 75 PG 26/600 column (GE Healthcare, Chicago, IL, USA) in a buffer containing 20 mM HEPES-NaOH (pH 7.5), 150 mM NaCl, 5 mM DTT, 10% (w/v) glycerol at pH 7.5. The efficiency of the reaction was evaluated as the ratio between the concentration of CaM and dansyl calculated from the absorbance at 280 and 333 nm, respectively.

### Time-resolved fluorescence measurements

Dansyl fluorescence was excited at 355 nm by the doubled output of the Ti:sapphire laser, and the emission was isolated at 540 nm using the combination of a monochromator and a dielectric long-pass filter with a cut-off wavelength of 520 nm (Chroma, USA) placed in front of its input slit. The emission signal was collected and processed by the SPC150 TCSPC module (Becker-Hickl, Germany) with a fast-timing microchannel-plate PMT (Hamamatsu, Japan). The experimental decays were deconvolved using the model-independent maximum entropy method (MEM)^77,78^. Samples were placed in a thermostatic holder, and all experiments were performed at 23 °C in a buffer containing 20 mM HEPES-NaOH (pH 7.5), 150 mM NaCl, 1 mM CaCl_2_, 1 mM TCEP. The DANS-CaM, DAPK2, and 14-3-3γ concentrations were 25, 30, and 100 μM, respectively.

### Statistics and reproducibility

Results from the FP assay (Fig. 1b and Supplementary Fig. S12a), dephosphorylation assay (Fig. 6 and Supplementary Fig. S11b), and enzyme-activity measurements (Supplementary Fig. S6) are represented as means ± SD from three replicates as indicated in the figure legend. Results from the DSF measurements (Supplementary Fig. S1) are represented as means ± SD from seven replicates. Statistical analysis was performed using Graph-Pad Prism 8.4. Student’s *t*-test was used for comparison of relative changes of samples (ns, non-significant *P* > 0.05; *P ≤ 0.05; **P ≤ 0.01; ***P ≤ 0.001; ****P ≤ 0.0001).

### Reporting summary

Further information on research design is available in the Nature Research Reporting Summary linked to this article.

## Supplementary information

Supplementary Information

Description of Supplementary Files

Supplementary Data 1

Reporting Summary

Final Revisions Checklist

Validation Report

Validation Report

## Data Availability

The authors declare that all data supporting the findings of this study are available within the article and its Supplementary Information file. Crystallography data have been deposited in the RCSB PDB with the accession codes: 7A6R and 7A6Y. All source data underlying the graphs presented in the main and supplementary figures are made available in Supplementary Data 1. Any remaining information can be obtained from the corresponding author upon reasonable request.
