# Supplementary material for: 14-3-3 proteins inactivate DAPK2 by promoting its dimerization and protecting key regulatory phosphosites
Source: Commun Biol. 2021 Aug 19;4:986. doi: 10.1038/s42003-021-02518-y (PMC8376927; doi:10.1038/s42003-021-02518-y)
Supplement: Supplementary file 1 — Supplementary Information [file 42003_2021_2518_MOESM1_ESM.pdf]

Supplementary information for

14-3-3 proteins inactivate DAPK2 by promoting its dimerization and protecting key regulatory phosphosites

Matej Horvath[1,2], Olivia Petrvalska[1,2], Petr Herman[3], Veronika Obsilova[2,*], and Tomas Obsil[1,2,*]

[1] Department of Physical and Macromolecular Chemistry, Faculty of Science, Charles University, Prague, Czech Republic
[2] Department of Structural Biology of Signaling Proteins, Division BIOCEV, Institute of Physiology of the Czech Academy of Sciences, 252 50 Vestec, Czech Republic
[3] Institute of Physics, Faculty of Mathematics and Physics, Charles University, Prague, 12116 Prague, Czech Republic

*Corresponding authors: obsil@natur.cuni.cz (T.O.), veronika.obsilova @fgu.cas.cz (V.O.)

Content:

	**Title**	**Page**
Table S1	Structural parameters determined from SAXS data	2
Table S2	Unique intermolecular cross-links between $DAPK2_{RNTD}$ and 14-3-3γ in disuccinimidyl glutarate (DSG).	3
Table S3	Summary of time-resolved DANS-CaM fluorescence measurements.	4
Table S4	Oligonucleotide sequences	5
Fig. S1	Stabilization of the interaction between ctDAPK2-pT^{369} and 14-3-3γΔC by FC-A	6
Fig. S2	Comparison of the 14-3-3γ:ctDAPK2-pT^{369} and 14-3-3γ:ctDAPK2-pT^{369}:FC-A complexes	7
Fig. S3	Detection of DAPK2 Ser^{318} autophosphorylated peptides by FT-ICR mass spectrometry	8
Fig. S4	Detection of DAPK2 Thr^{369} autophosphorylated peptides by FT-ICR mass spectrometry	9
Fig. S5	Example of MS/MS data from the analysis of phosphorylated peptides.	10
Fig. S6	DAPK2 kinase activity measurements	11
Fig. S7	SEC-SAXS analysis of the 14-3-3γ:$DAPK2_{RNTD}$ complex	12
Fig. S8	SAXS-based rigid-body modeling of the 14-3-3γ:$DAPK2_{RNTD}$ complex	13
Fig. S9	Example of MS/MS data from XL-MS experiments	15
Fig. S10	Time-resolved dansyl fluorescence measurements	16
Fig. S11	14-3-3γ slows down DAPK2 dephosphorylation.	17
Fig. S12	14-3-3γ binds to the motif containing Ser^{318} from the AID.	19
Supplementary References		20

Supplementary Table S1. Structural parameters determined from SAXS data.

Sample	R_g (Å)[e]	R_g (Å)[f]	D_{max} (Å)	V_P[g] (nm^3)	M_w[h] (kDa)	M_w[i] (kDa)
$DAPK2_{RNTD:}$14-3-3γ (2:2)[a]	40.9 ± 0.1	41.1 ± 0.1	133	246	147	143.4
$DAPK2_{RNTD}$ (1)[a,c]	27.0 ± 0.1	27.6 ± 0.1	94	60	44	43.2[j]
$DAPK2_{RNTD}$ (2)[a,d]	28.6 ± 0.1	29.2 ± 0.1	96	70	56	43.2[j]
14-3-3γ[b]	29.6 ± 0.1	29.8 ± 0.1	89	87	58	57.0

[a] SEC-SAXS measurements
[b] Batch measurement (protein concentration 3.4 $mg.mL^{-1}$)
[c] Based on frames from the right side of the elution peak from SEC
[d] Based on frames from the left side of the elution peak from SEC
[e] Calculated using the Guinier approximation
[f] Calculated using GNOM[1]
[g] Excluded volume of the hydrated particle (Porod volume)
[h] Molecular weight estimate based on a consensus Bayesian assessment method[2]
[i] Theoretical molecular weights
[j] Value of the $DAPK2_{RNTD}$ protomer

Supplementary Table S2. Unique intermolecular cross-links between DAPK2$_{RNTD}$ and 14-3-3γ in disuccinimidyl glutarate (DSG).

	Crosslinked peptides		Crosslinked residues		Mass	Error (ppm)
	DAPK2	14-3-3γ	DAPK2	14-3-3γ		
DSG						
1.	8-18	118-133	S8	K127	3534.71	-0.35
2.	8-18	118-133	K15	S119	3534.71	-0.35
3.	8-18	118-133	K15	K125	3534.71	-0.35
4.	132-143	199-217	Y139	S215	3626.74	-0.08
5.	132-143	199-217	T142	S215	3626.74	-0.69
6.	308-312	199-227	K308	S215	4106.03	0.19
7.	315-334	11-28	S331	Y20	4555.27	-0.51
8.	331-345	228-247	S331	T231	4125.91	0.43
9.	335-339	20-42	K335	Y20	3403.62	-0.95
10.	335-339	20-42	K335	K28	3403.62	-0.95
11.	335-345	199-227	K335	S215	4774.39	0.44
12.	359-364	62-77	K359	K69	2522.32	0.22
13.	359-364	62-77	K359	S71	2522.32	0.18
14.	359-364	199-227	K359	S215	4118.08	0.01
15.	359-364	199-227	K359	K217	4118.08	0.03
16.	359-364	199-227	K359	S219	4118.08	-0.11

Supplementary Table S3. Summary of time-resolved DANS-CaM fluorescence measurements.

Sample	τ_{mean}[a,b] (ns)	ϕ_1 (ns)	β_1[c]	ϕ_2 (ns)	β_2	ϕ_3 (ns)	β_3	ϕ_4[d] (ns)	β_4	ϕ_5 (ns)	β_5	r_0
DANS-CaM alone	15.9	1.3	0.06	2.3	0.07	8.1	0.17					0.30
14-3-3γ	15.9	1.0	0.06	2.9	0.10	9.3	0.15					0.31
$DAPK2_{RNTD}$	20.5			2.4	0.03	13.6	0.06	52	0.22			0.31
$DAPK2_{RNTD}$/14-3-3γ	19.6	0.47	0.02	3.6	0.07	20.6	0.07	95	0.14			0.31
$DAPK2_{RNTD}S^{318}A$	18.2			2.3	0.02	8.5	0.06	39.6	0.15	108[e]	0.09	0.32
$DAPK2_{RNTD}S^{318}A$/14-3-3γ	18.1	0.5	0.03	3.3	0.02	12.9	0.07	57	0.19	>150[e]	0.02	0.33

[a]Mean lifetimes were calculated as $\tau_{mean}=\Sigma_i f_i \tau_i$, where f_i is an intensity fraction of the *i*-th lifetime component τ_i.
[b]SD = ± 0.1 ns
[c]The emission anisotropies $r(t)$ were analyzed for a series of exponentials using a model-independent maximum entropy method without setting any assumptions about the shape of the anisotropy decay and the correlation time distribution [3,4], $r(t) = \Sigma_i \beta_i' \exp(-t/\phi_i')$, where the amplitudes β_i' represent the distribution of the correlation times ϕ_i'. Values β_i represent peaks of the distribution positioned at the correlation times ϕ_i.
[d]SD = ± 7 ns
[e]The very long lifetime component likely reflects minor aggregation.

Supplementary Table S4. Oligonucleotide sequences.

Oligonucleotide	Sequence
Set 1 for *DAPK2* in pHGT2	
pHGT2_fw (*BamH*I)	ACGCGGATCCTTCCAGGCCTCAATGAGG
pHGT2_rev (*Not*1)	CGTCGTCCTTGGCGGCCGCCTAGGAGGTGCTGCTCCTCC
Set 2 for mutating C-terminus of *DAPK2*	
S367R, S368N, S370D_fw	CACGGAGGAGGAGGAACACCGACTAGGCGGCCGC
S367R, S368N, S370D_rev	GCGGCCGCCTAGTCGGTGTTCCTCCTCCTCCGTG
Set 3 for *DAPK2* S318A mutation	
S318A_fw	GCGGTGGAAGCTTGCCTTCAGCATCGTGTC
S318A_rev	GACACGATGCTGAAGGCAAGCTTCCACCGC

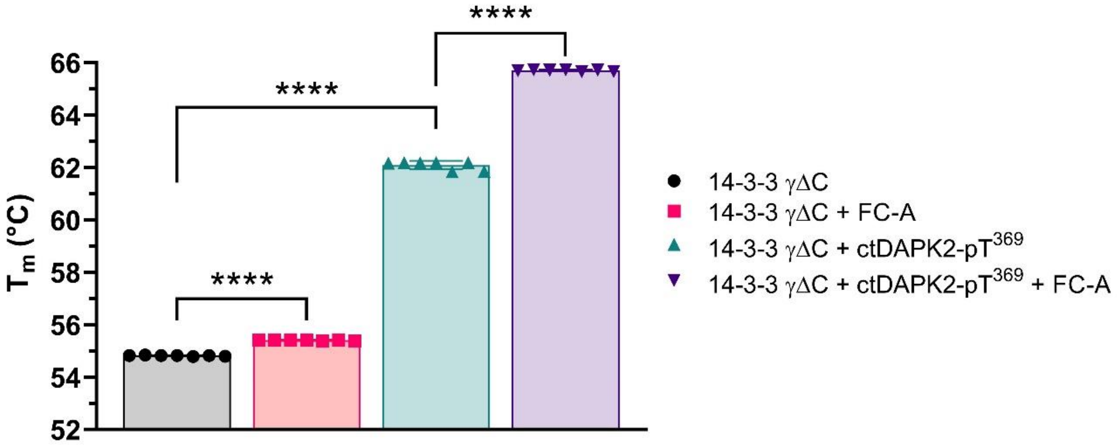

Supplementary Figure S1. Stabilization of the interaction between ctDAPK2-pT^{369} and 14-3-3γΔC by FC-A. DSF was used to test the thermal stability of 7.5 µM 14-3-3γΔC with and without 200 µM ctDAPK2-pT^{369} (sequence RRRSSpTS), representing the DAPK2 C-terminal 14-3-3 binding motif, and/or 500 µM Fusicoccin-A (FC-A). As noted, ctDAPK2-pT^{369} binding significantly increased the melting temperature (T_m) of 14-3-3γΔC from 54.8 ± 0.1 to 62.1 ± 0.2 °C. The presence of FC-A stabilized the 14-3-3γΔC:ctDAPK2-pT^{369} complex, as shown by the increase in T_m to 65.7 ± 0.1 °C. Error bars represent the standard deviation of seven independent measurements. Asterisks represent significant differences according to Student's t-test comparing selected experiments (****, $P \leq 0.0001$).

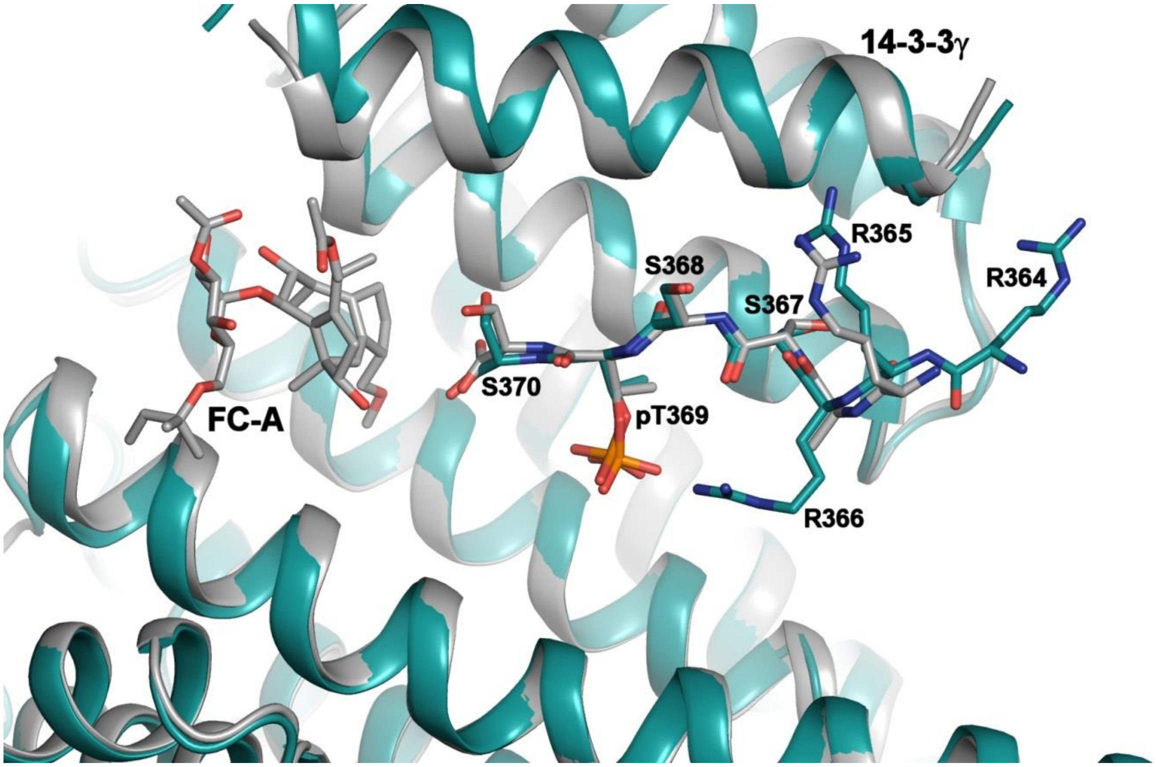

Supplementary Figure S2. Comparison of the 14-3-3γ:ctDAPK2-pT^{369} and 14-3-3γ:ctDAPK2-pT^{369}:FC-A complexes. Superimposition of the 14-3-3γ:ctDAPK2-pT^{369} (shown in cyan) and 14-3-3γ:ctDAPK2-pT^{369}:FC-A (shown in gray) complexes shows the similar position and interactions of the ctDAPK2-pT^{369} peptide within the ligand binding groove of 14-3-3γ.

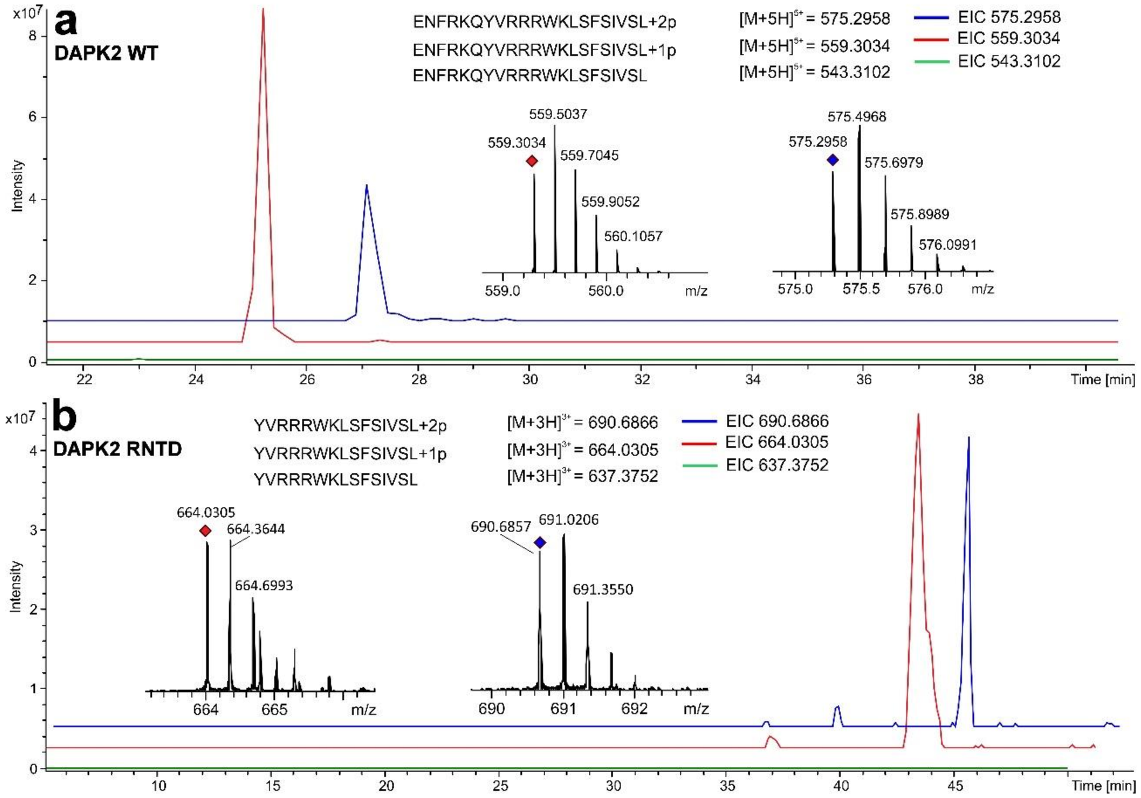

Supplementary Figure S3. Detection of DAPK2 Ser^{318} autophosphorylated peptides by FT-ICR mass spectrometry. a Extract ion chromatograms (EIC) of phosphorylated E^{304}NFRKQYVRRRWKLSFSIVSL^{324} peptides of DAPK2 WT. The EIC of doubly and singly phosphorylated peptides are shown in blue (m/z 575.2958 (5+)) and red (m/z 559.3034 (5+)), respectively. The green line represents the EIC of the non-phosphorylated form of the same peptide observed at m/z 543.3102 (5+). The inset shows the zoomed-in, high-resolution MS spectra of phosphorylated peptides. **b** Extract ion chromatograms of the phosphorylated Y^{310}VRRRWKLSFSIVSL^{324} peptides of DAPK2$_{RNTD}$. The EIC of doubly and singly phosphorylated peptides are shown in blue (m/z 690.6866 (3+)) and red (m/z 664.0305 (3+)), respectively. The green line represents the EIC of the non-phosphorylated form of the same peptide observed at m/z 637.3752 (3+). The inset shows the zoomed-in, high-resolution MS spectra of phosphorylated peptides.

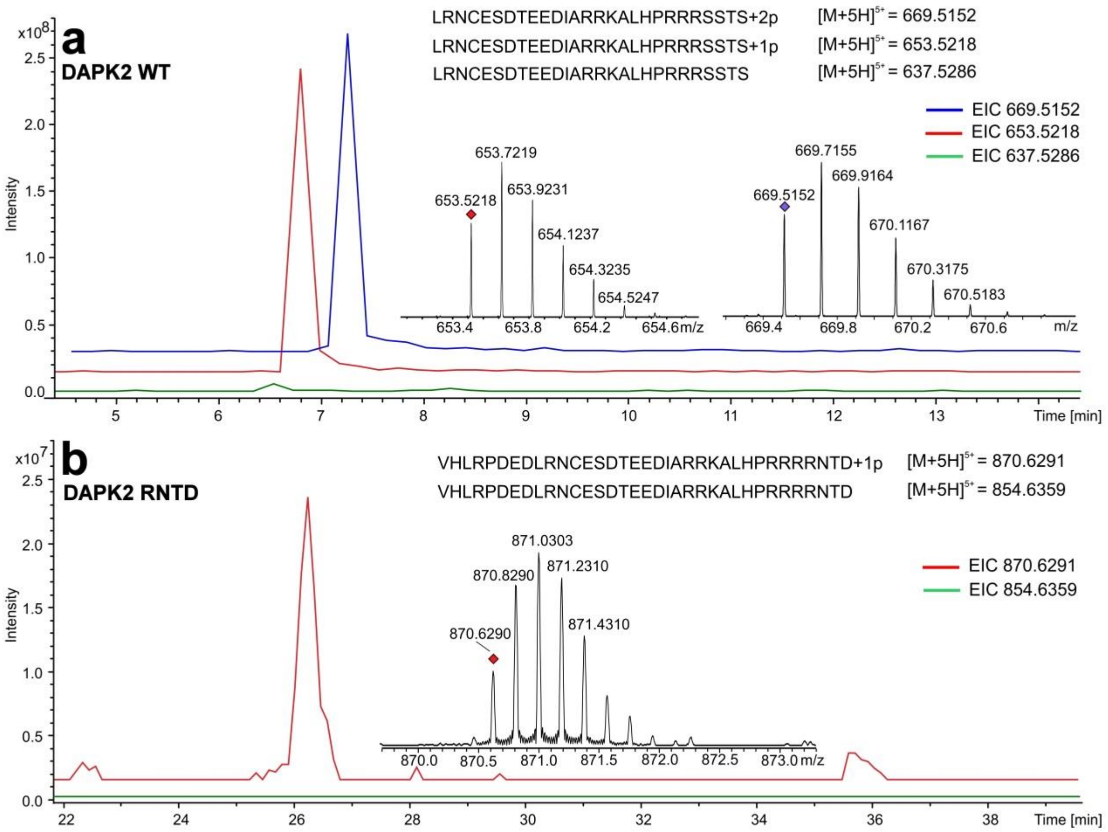

Supplementary Figure S4. Detection of DAPK2 Thr^{369} autophosphorylated peptides by FT-ICR mass spectrometry. a Extract ion chromatograms (EIC) of phosphorylated L^{344}RNCESDTEEDIARRKALHPRRRSSTS^{370} peptides of DAPK2 WT. The EIC of doubly and singly phosphorylated peptides are shown in blue (m/z 669.5152 (5+)) and red (m/z 653.5218 (5+)), respectively. The green line represents the EIC of the non-phosphorylated form of the same peptide observed at m/z 637.5286 (5+). The inset shows the zoomed-in, high-resolution MS spectra of phosphorylated peptides. **b** Extract ion chromatogram of the phosphorylated V^{336}HLRPDEDLRNCESDTEEDIARRKALHPRRRRNTD^{370} peptide of DAPK2$_{RNTD}$. The EIC of singly phosphorylated peptide is shown in red (m/z 870.6291 (5+)). The green line represents the EIC of the non-phosphorylated form of the same peptide observed at m/z 854.6359 (5+). The inset shows the zoomed-in, high-resolution MS spectrum of the phosphorylated peptide.

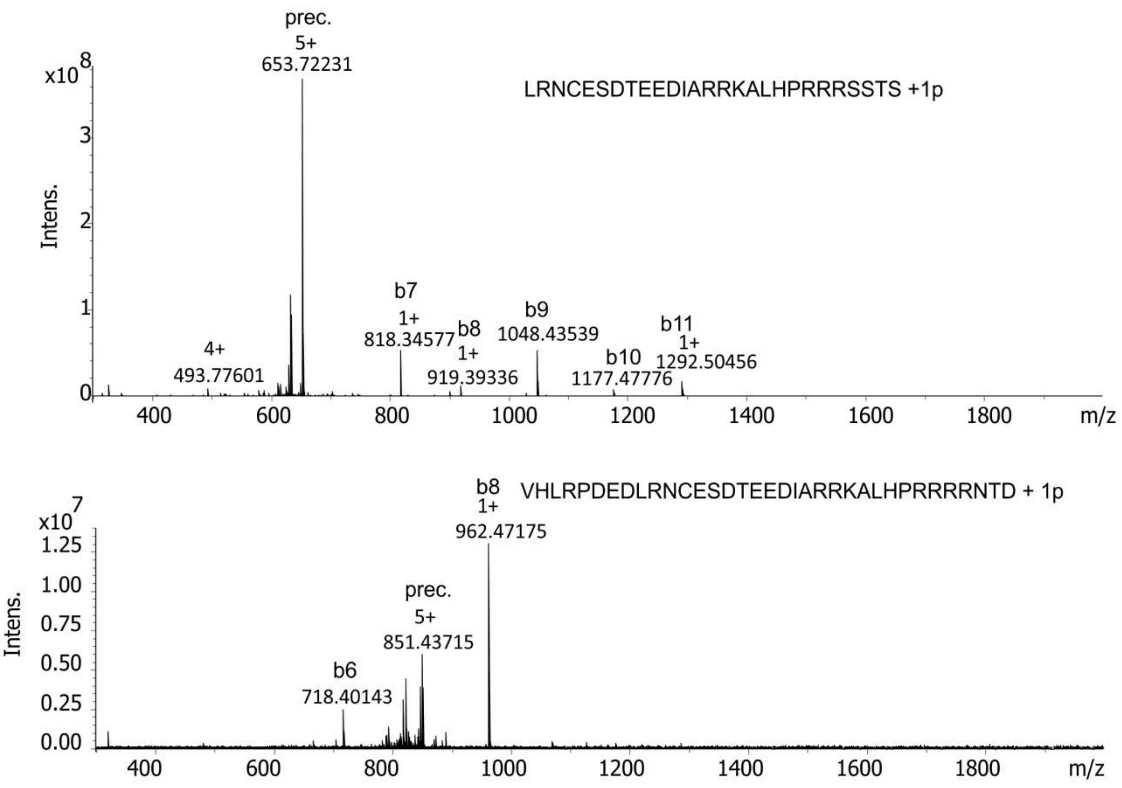

Supplementary Figure S5. Example of MS/MS data from the analysis of phosphorylated peptides. Collisionally induced fragmentation spectra of phosphorylated peptides L^{344}–S^{370} and V^{336}–D^{370} of DAPK2 WT and $DAPK2_{RNTD}$, respectively.

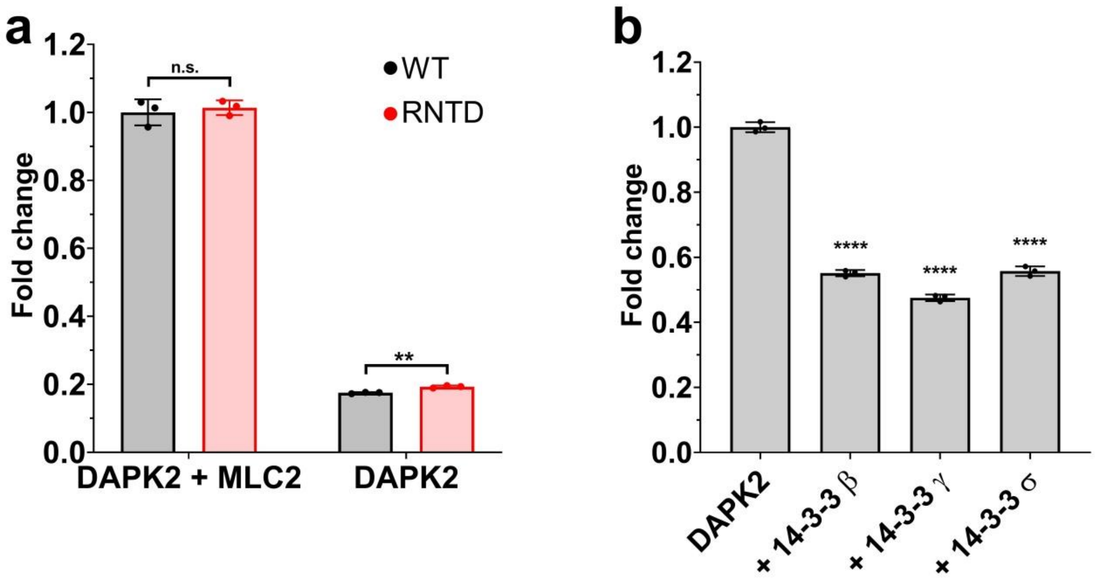

Supplementary Figure S6. DAPK2 kinase activity measurements. a Comparison of the kinase activity of DAPK2$_{RNTD}$ and DAPK2 WT. The specific activity of DAPK2 WT with the peptide containing the N-terminal part of the Myosin regulatory light chain, sequence KKRAARATSNVFA, as a substrate was 30.9 ± 0.3 nmol.min^{-1}.mg^{-1} (labeled as DAPK2 + MLC). Specific activity was calculated by quantifying the ADP produced using the ADP-Glo^{TM} assay (Promega, USA), according to the manufacturer's instructions. The kinase activity observed in the absence of MLC peptide (labeled as DAPK2) represents the ADP production in the absence of MLC peptide. Error bars represent the standard deviation of three independent measurements. Asterisks represent significant differences (**, $P \leq 0.01$) according to Student's t-test comparing DAPK2$_{RNTD}$ with DAPK2 WT in the presence and absence of MLC2 substrate. **b** 14-3-3 proteins suppress the kinase activity of DAPK2$_{RNTD}$ with the MLC peptide as a substrate. The relative activities of DAPK2$_{RNTD}$ in the presence of selected human 14-3-3 isoforms were normalized to the relative activity of DAPK2$_{RNTD}$ alone. Error bars represent the standard deviation of three independent measurements. Asterisks represent significant differences (****, $P \leq 0.0001$) according to Student's t-test comparing DAPK2$_{RNTD}$ alone with DAPK2$_{RNTD}$ in the presence of 14-3-3.

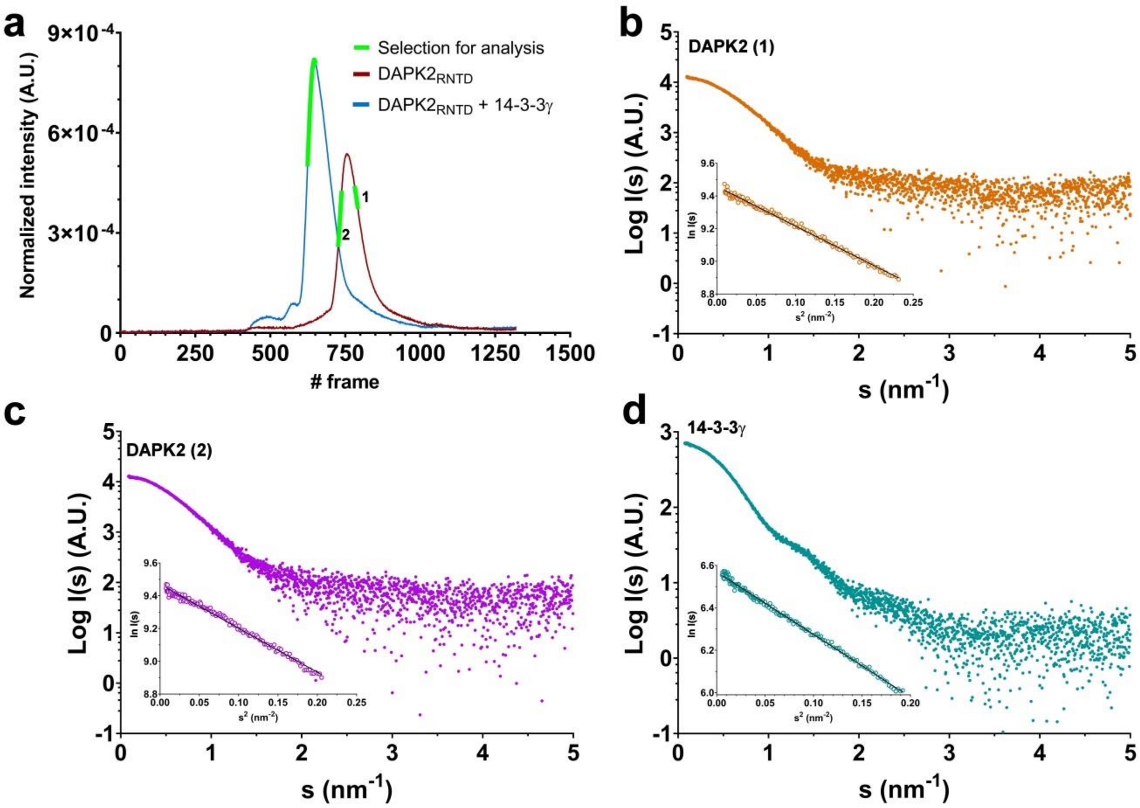

Supplementary Figure S7. SEC-SAXS analysis of the DAPK2$_{RNTD}$:14-3-3γ complex. a SEC-SAXS elution profiles of DAPK2$_{RNTD}$ and the mixture of 14-3-3γ and DAPK2$_{RNTD}$. The regions that were used for further analysis are shown in green. **b-d** Scattering intensity as a function of the scattering vector *s* ($s = 4\pi\sin(\theta/\lambda)$, where 2θ is the scattering angle, and λ is the wavelength) of the DAPK2$_{RNTD}$ (regions 1 and 2) and 14-3-3γ. The insets show the Guinier plots of the scattering data.

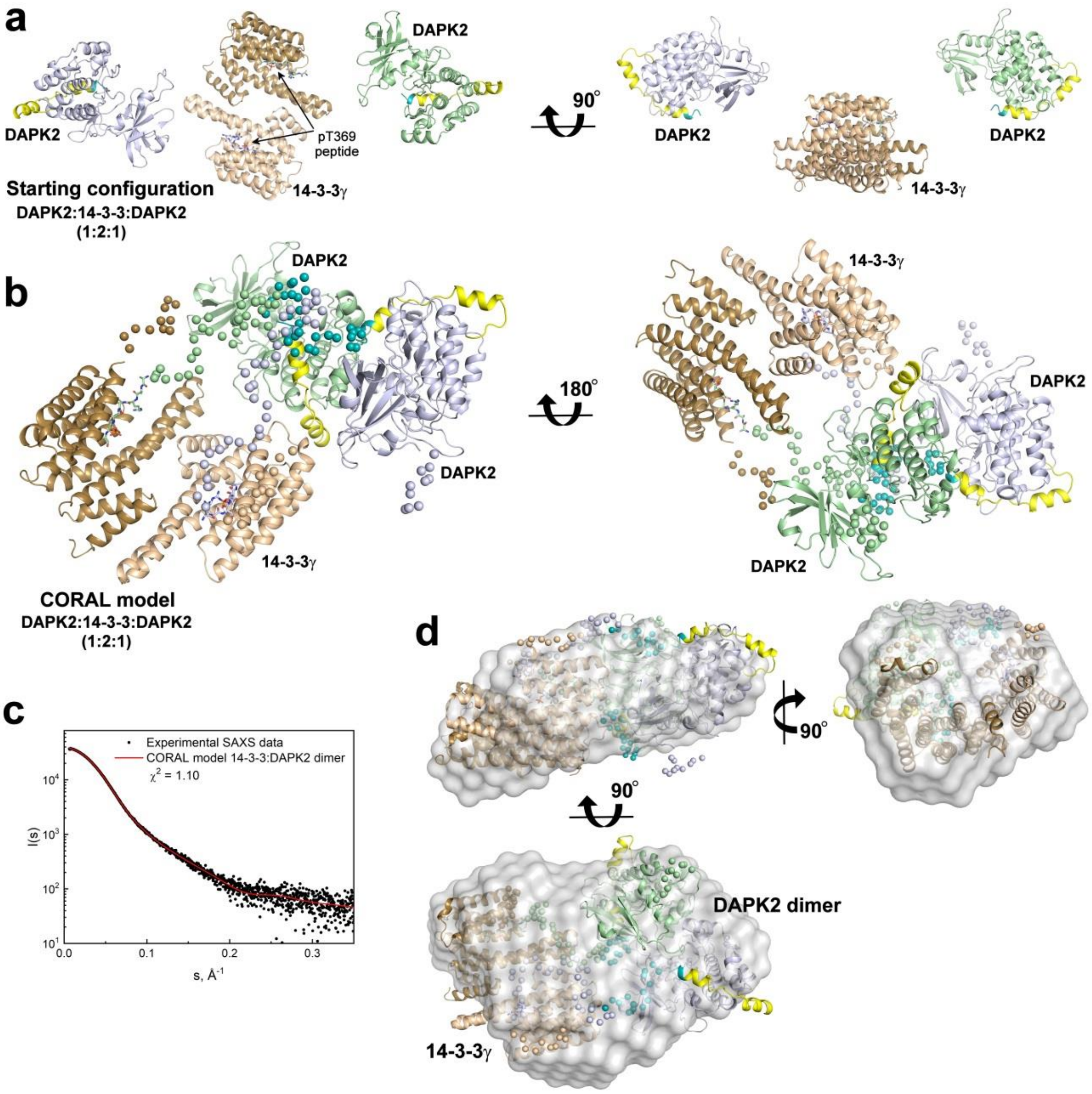

Supplementary Figure S8. SAXS-based modeling of the DAPK2$_{\mathbf{RNTD}}$:14-3-3γ complex. a Starting conformation of the rigid-body modeling of the DAPK2$_{RNTD}$:14-3-3γ complex, which was modeled as two isolated DAPK2 protomers bound to the 14-3-3γ dimer via their C-terminal pT^{369} motifs. **b** The best-scoring CORAL model based on the starting conformation is shown in panel A. The unstructured segments missing in the crystal structures were modeled as dummy residue chains (shown as spheres). The AID and CBD of DAPK2 are colored in yellow and cyan, respectively. The ctDAPK2-pT^{369} peptide is shown as sticks. As noted, both DAPK2 protomers are located close to each other, thus suggesting that the DAPK2$_{RNTD}$:14-3-3γ complex with 2:2 stoichiometry consists of the DAPK2 dimer bound to the 14-3-3γ dimer. **c** Experimental

scattering curve of the $DAPK2_{RNTD}$:14-3-3γ complex superimposed with the calculated curve of the best-scoring CORAL model of the DAPK2 dimer:14-3-3γ dimer complex (shown in red). **d** *Ab initio* shape reconstruction of the $DAPK2_{RNTD}$:14-3-3γ complex (gray envelope) calculated from SAXS data with a superimposed CORAL model of the dimer:dimer complex.

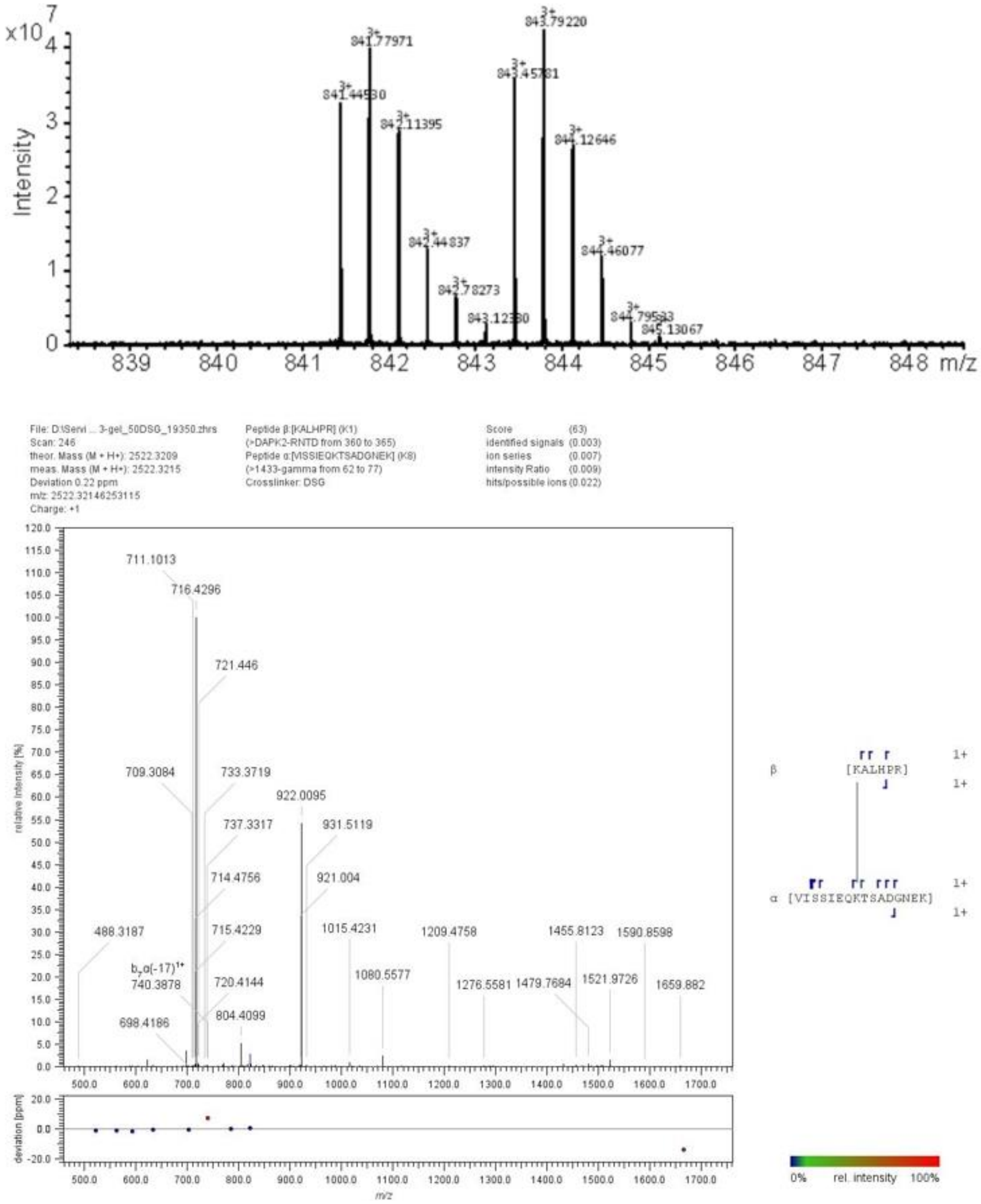

Supplementary Figure S9. Example of MS/MS data from XL-MS experiments. MS spectrum of the intermolecular peptide KALHPR–VISSIEQKTSADGNEK (DAPK2$_{RNTD}$–14-3-3γ) at m/z 841.4453 (3+) cross-linked by the DSG reagent (top). The ion duplet represents a peptide modified with DSG-H6 and DSG-D6 in a 1:1 ratio. The fragment spectrum (bottom) of the cross-linked peptide was matched using the StavroX software.

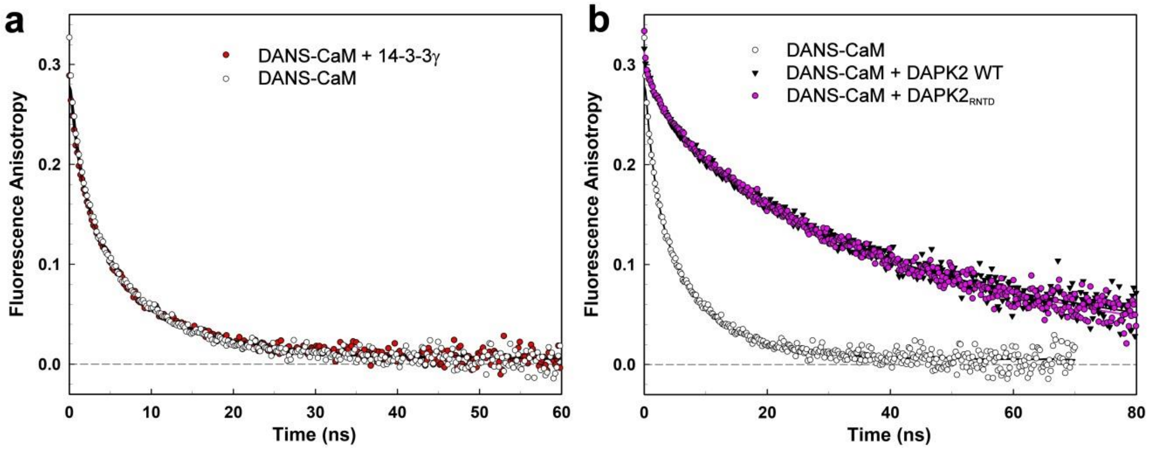

Supplementary Figure S10. Time-resolved dansyl fluorescence measurements. a Fluorescence anisotropy decays of free dansyl-Ca^{2+}/CaM with (red circles) and without (open circles) 14-3-3γ. **b** Fluorescence anisotropy decays of free dansyl-Ca^{2+}/CaM (open circles) and dansyl-Ca^{2+}/CaM with DAPK2 WT (black triangles) and $DAPK2_{RNTD}$ (violet circles).

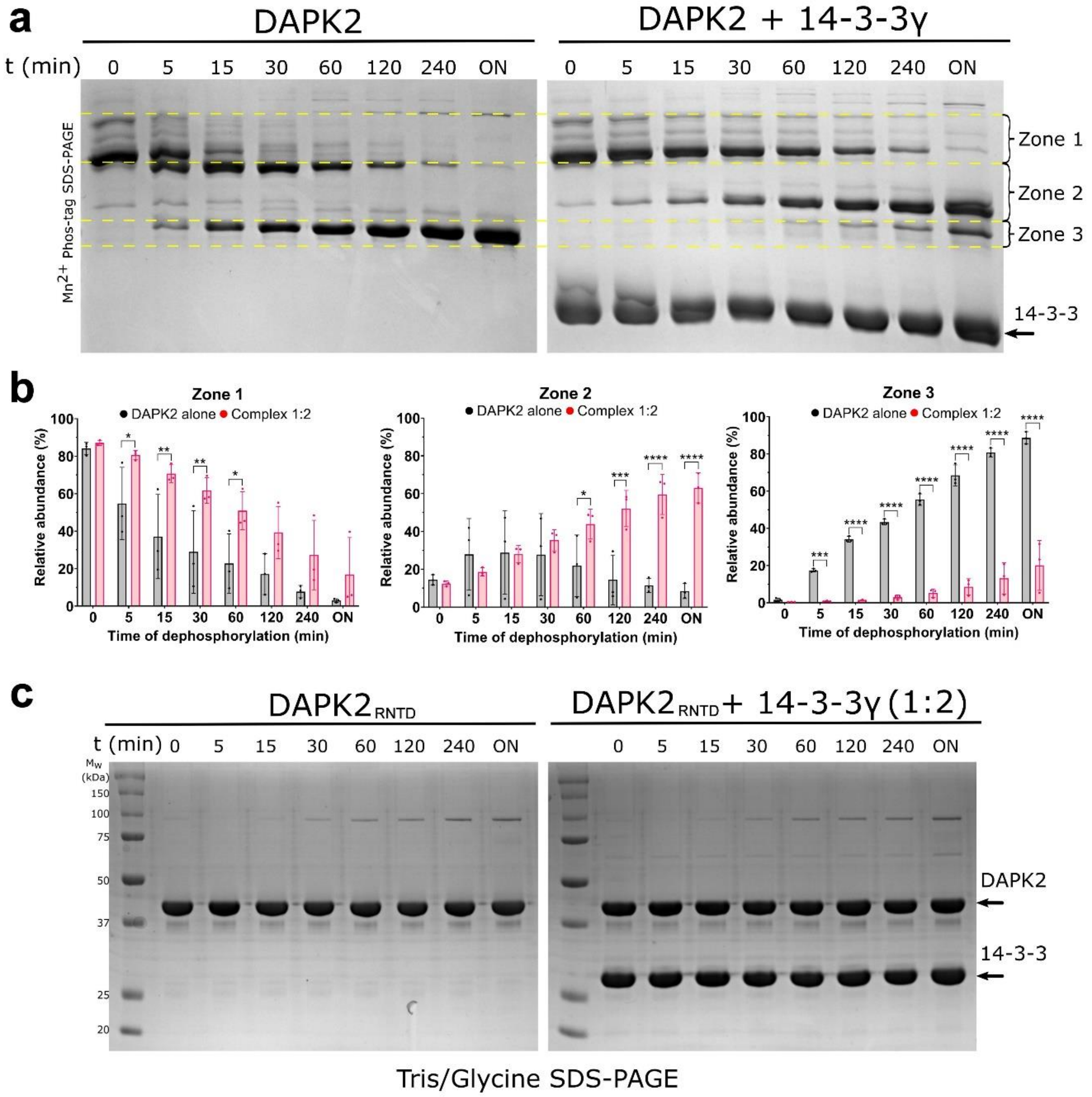

Supplementary Figure S11. 14-3-3γ slows down DAPK2 dephosphorylation. a Representative Mn^{2+} Phos-tag SDS-PAGE gels showing the time-dependent dephosphorylation of $DAPK2_{RNTD}$ by type 1 protein phosphatase (PP1) with and without 14-3-3γ. $DAPK2_{RNTD}$ (6 nmol) was incubated with 6 units of PP1 with and without 14-3-3γ (12 nmol) at 21 °C. The reaction was stopped at specific time points and analyzed by 100 µM Mn^{2+}-Phos-tag™ 10% SDS-PAGE stained with Coomassie G-250. Yellow dashed lines denote the borders of DAPK2 phospho-variants zones that were quantified (Zone 1, highly phosphorylated $DAPK2_{RNTD}$; Zone 2, partly phosphorylated $DAPK2_{RNTD}$; Zone 3, dephosphorylated $DAPK2_{RNTD}$). **b** Relative abundances of

$DAPK2_{RNTD}$ phospho-variants after dephosphorylation by PP1 with and without 14-3-3γ based on Mn^{2+} Phos-tag SDS-PAGE. The relative abundances of $DAPK2_{RNTD}$ phospho-variants were calculated as the ratio between the intensity of a specific zone and the sum of intensities from all three zones within the same lane expressed as percentage. Error bars represent the standard deviation of three independent experiments. Asterisks represent significant differences according to Student's t-test comparing DAPK2 dephophorylation with and without 14-3-3γ (*, $P \leq 0.05$; **, $P \leq 0.01$; ***, $P \leq 0.001$; ****, $P \leq 0.0001$). **c** Purity and integrity of samples used in the dephosphorylation experiments. The samples were resolved by 12% Tris/Glycine SDS-PAGE and stained with Coomasie G-250 dye.

a

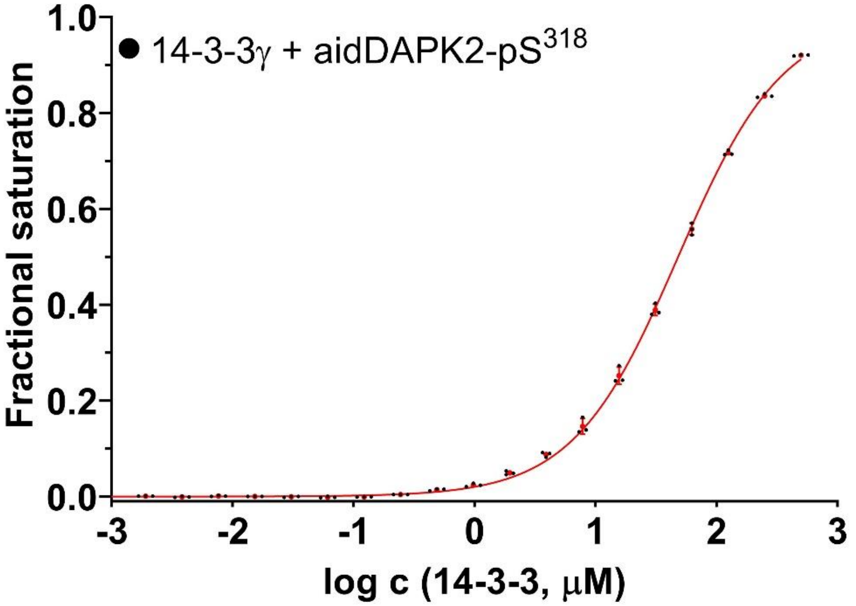

b

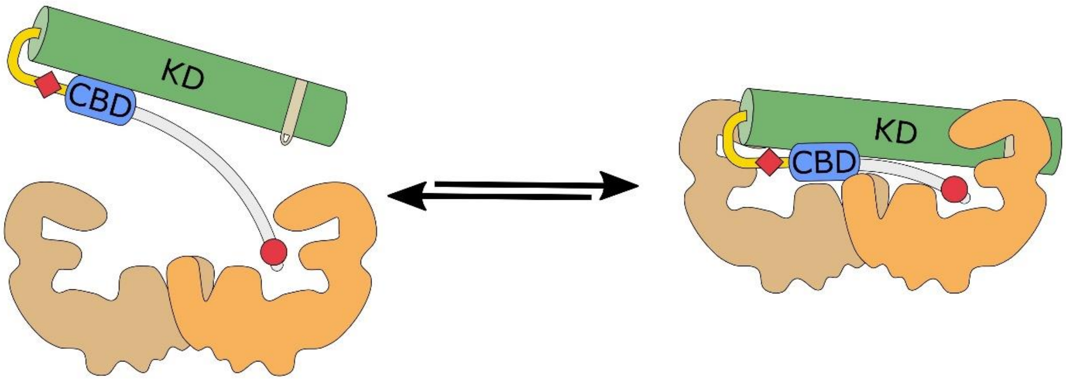

Supplementary Figure S12. 14-3-3γ binds to the motif containing Ser^{318} from the AID. a Fluorescence polarization (FP) measurements of FAM-labeled aidDAPK-pS^{318} peptide (sequence FAM-RRRWKLpSFSIV) titrated by 14-3-3γ. The binding affinity was determined by fitting the FP data to a one-site-binding model, with K_D of 48 ± 1 μM. All data points are the means ± SD of three replicates. **b** Alternative variants of the inactive DAPK2$_{RNTD}$:14-3-3γ complex with a 1:2 stoichiometry. The high affinity binding motif pThr^{369} (red circle) functions as a primary 14-3-3 binding site, whereas the Ser^{318}-containing motif (red diamond) may play the role of a secondary 14-3-3 binding site.

Supplementary References

1. Svergun, D.I. Determination of the Regularization Parameter in Indirect-Transform Methods Using Perceptual Criteria. *Journal of Applied Crystallography* **25**, 495-503 (1992).
2. Hajizadeh, N.R., Franke, D., Jeffries, C.M. & Svergun, D.I. Consensus Bayesian assessment of protein molecular mass from solution X-ray scattering data. *Sci Rep* **8**, 7204 (2018).
3. Vecer, J. & Herman, P. Maximum Entropy Analysis of Analytically Simulated Complex Fluorescence Decays. *Journal of Fluorescence* **21**, 873-881 (2011).
4. Bryan, R.K. Maximum-Entropy Analysis of Oversampled Data Problems. *European Biophysics Journal* **18**, 165-174 (1990).
