# Supplementary material for: 14-3-3 proteins inactivate DAPK2 by promoting its dimerization and protecting key regulatory phosphosites
Source: Commun Biol. 2021 Aug 19;4:986. doi: 10.1038/s42003-021-02518-y (PMC8376927; doi:10.1038/s42003-021-02518-y)
Supplement: Supplementary file 2 — Description of Supplementary Files [file 42003_2021_2518_MOESM2_ESM.pdf]

Description of Additional Supplementary Files

File name: Supplementary Data 1

Description: Raw data for Figures 1b, 3-6, and Supplementary Figures S1, S6, S7, S8c, S10, S11b, and S12a.
