# Supplementary material for: 14-3-3 proteins inactivate DAPK2 by promoting its dimerization and protecting key regulatory phosphosites
Source: Commun Biol. 2021 Aug 19;4:986. doi: 10.1038/s42003-021-02518-y (PMC8376927; doi:10.1038/s42003-021-02518-y)
Supplement: Supplementary file 7 — Validation Report [file 42003_2021_2518_MOESM7_ESM.pdf]

Full wwPDB X-ray Structure Validation Report (i)

CONFIDENTIAL VALIDATION REPORT

Mar 17, 2021 – 12:35 pm GMT

PDB ID	:	7A6Y
Title	:	Structure of 14-3-3 gamma in complex with DAPK2 peptide stabilized by FC-A
Deposited on	:	2020-08-27
Resolution	:	2.50 Å(reported)

This is a Full wwPDB X-ray Structure Validation Report.

This report is produced by the wwPDB biocuration pipeline after annotation of the structure.

We welcome your comments at *validation@mail.wwpdb.org*
A user guide is available at
https://www.wwpdb.org/validation/2017/XrayValidationReportHelp
with specific help available everywhere you see the (i) symbol.

The following versions of software and data (see references (i)) were used in the production of this report:

MolProbity	:	4.02b-467
Mogul	:	1.8.5 (274361), CSD as541be (2020)
Xtriage (Phenix)	:	1.13
EDS	:	2.17.1
buster-report	:	1.1.7 (2018)
Percentile statistics	:	20191225.v01 (using entries in the PDB archive December 25th 2019)
Refmac	:	5.8.0158
CCP4	:	7.0.044 (Gargrove)
Ideal geometry (proteins)	:	Engh & Huber (2001)
Ideal geometry (DNA, RNA)	:	Parkinson et al. (1996)
Validation Pipeline (wwPDB-VP)	:	2.17.1

1 Overall quality at a glance (i)

The following experimental techniques were used to determine the structure:
X-RAY DIFFRACTION

The reported resolution of this entry is 2.50 Å.

Percentile scores (ranging between 0-100) for global validation metrics of the entry are shown in the following graphic. The table shows the number of entries on which the scores are based.

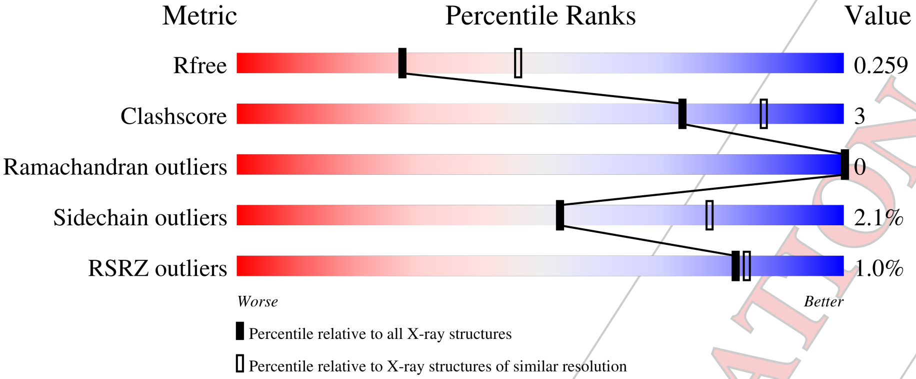

Metric	Whole archive (#Entries)	Similar resolution (#Entries, resolution range(Å))
R_{free}	130704	4661 (2.50-2.50)
Clashscore	141614	5346 (2.50-2.50)
Ramachandran outliers	138981	5231 (2.50-2.50)
Sidechain outliers	138945	5233 (2.50-2.50)
RSRZ outliers	127900	4559 (2.50-2.50)

The table below summarises the geometric issues observed across the polymeric chains and their fit to the electron density. The red, orange, yellow and green segments of the lower bar indicate the fraction of residues that contain outliers for >=3, 2, 1 and 0 types of geometric quality criteria respectively. A grey segment represents the fraction of residues that are not modelled. The numeric value for each fraction is indicated below the corresponding segment, with a dot representing fractions <=5% The upper red bar (where present) indicates the fraction of residues that have poor fit to the electron density. The numeric value is given above the bar.

Mol	Chain	Length	Quality of chain
1	A	236	86% 8% • •
1	B	236	% 84% 7% 8%
1	C	236	2% 87% 8% 5%
1	D	236	% 83% 13% •
2	J	7	43% 43% 14%

Continued on next page...

CONFIDENTIAL VALIDATION REPORT

Continued from previous page...

Mol	Chain	Length	Quality of chain
2	K	7	86% 14%
2	L	7	71% 14% 14%
2	M	7	57% 14% 29%

CONFIDENTIAL VALIDATION REPORT

2 Entry composition (i)

There are 4 unique types of molecules in this entry. The entry contains 7579 atoms, of which 0 are hydrogens and 0 are deuteriums.

In the tables below, the ZeroOcc column contains the number of atoms modelled with zero occupancy, the AltConf column contains the number of residues with at least one atom in alternate conformation and the Trace column contains the number of residues modelled with at most 2 atoms.

- Molecule 1 is a protein called 14-3-3 protein gamma.

Mol	Chain	Residues	Atoms					ZeroOcc	AltConf	Trace
1	A	226	Total 1796	C 1129	N 310	O 348	S 9	0	0	0
1	B	217	Total 1724	C 1081	N 295	O 340	S 8	0	0	0
1	C	225	Total 1797	C 1125	N 310	O 353	S 9	0	0	0
1	D	229	Total 1795	C 1122	N 308	O 356	S 9	0	0	0

There are 8 discrepancies between the modelled and reference sequences:

Chain	Residue	Modelled	Actual	Comment	Reference
A	-1	GLY	-	expression tag	UNP P61981
A	0	HIS	-	expression tag	UNP P61981
B	-1	GLY	-	expression tag	UNP P61981
B	0	HIS	-	expression tag	UNP P61981
C	-1	GLY	-	expression tag	UNP P61981
C	0	HIS	-	expression tag	UNP P61981
D	-1	GLY	-	expression tag	UNP P61981
D	0	HIS	-	expression tag	UNP P61981

- Molecule 2 is a protein called DAPK2 C-terminal peptide.

Mol	Chain	Residues	Atoms					ZeroOcc	AltConf	Trace
2	J	6	Total 46	C 22	N 9	O 14	P 1	0	0	0
2	K	7	Total 45	C 22	N 7	O 15	P 1	0	0	0
2	L	6	Total 40	C 19	N 6	O 14	P 1	0	0	0
2	M	5	Total 41	C 19	N 8	O 13	P 1	0	0	0

CONFIDENTIAL VALIDATION REPORT

- Molecule 3 is FUSICOCCIN (three-letter code: FSC) (formula: $C_{36}H_{56}O_{12}$).

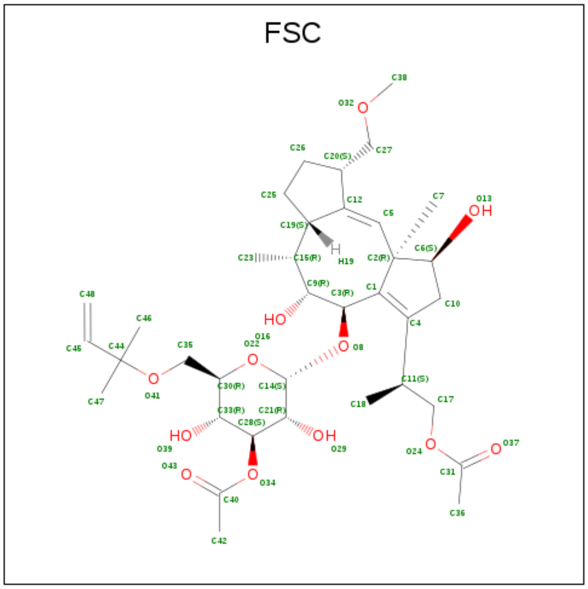

Mol	Chain	Residues	Atoms	ZeroOcc	AltConf
3	A	1	Total C O<br>48 36 12	0	0
3	C	1	Total C O<br>48 36 12	0	0
3	D	1	Total C O<br>48 36 12	0	0

- Molecule 4 is water.

Mol	Chain	Residues	Atoms	ZeroOcc	AltConf
4	A	67	Total O<br>67 67	0	0
4	B	39	Total O<br>39 39	0	0
4	C	29	Total O<br>29 29	0	0
4	D	12	Total O<br>12 12	0	0
4	J	2	Total O<br>2 2	0	0
4	L	1	Total O<br>1 1	0	0
4	M	1	Total O<br>1 1	0	0

CONFIDENTIAL VALIDATION REPORT

3 Residue-property plots (i)

These plots are drawn for all protein, RNA, DNA and oligosaccharide chains in the entry. The first graphic for a chain summarises the proportions of the various outlier classes displayed in the second graphic. The second graphic shows the sequence view annotated by issues in geometry and electron density. Residues are color-coded according to the number of geometric quality criteria for which they contain at least one outlier: green = 0, yellow = 1, orange = 2 and red = 3 or more. A red dot above a residue indicates a poor fit to the electron density (RSRZ > 2). Stretches of 2 or more consecutive residues without any outlier are shown as a green connector. Residues present in the sample, but not in the model, are shown in grey.

- Molecule 1: 14-3-3 protein gamma

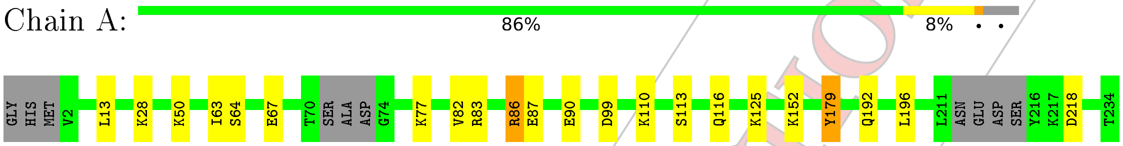

- Molecule 1: 14-3-3 protein gamma

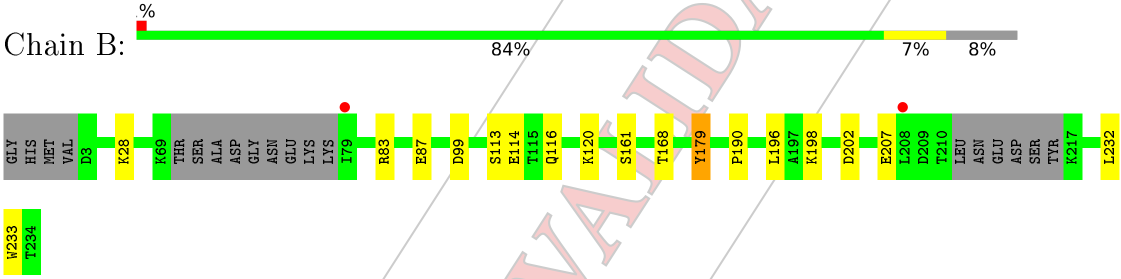

- Molecule 1: 14-3-3 protein gamma

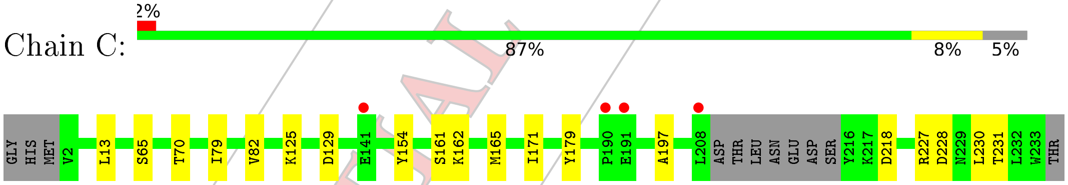

- Molecule 1: 14-3-3 protein gamma

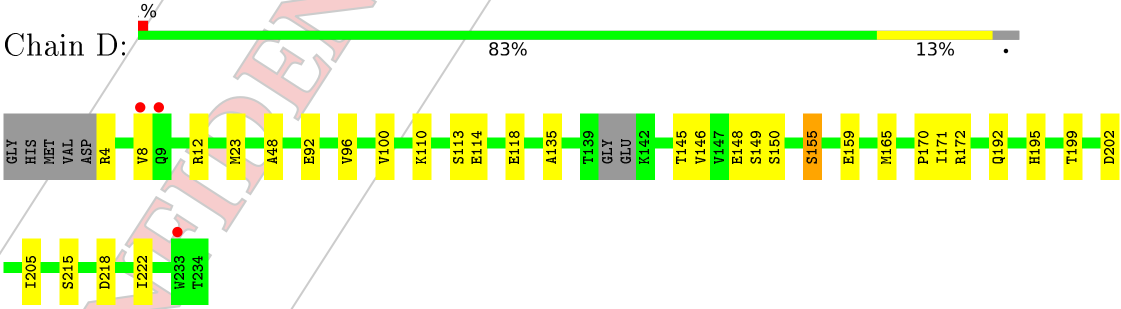

- Molecule 2: DAPK2 C-terminal peptide

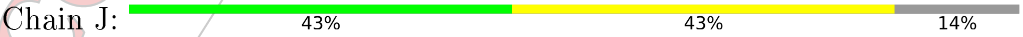

CONFIDENTIAL VALIDATION REPORT

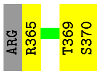

• Molecule 2: DAPK2 C-terminal peptide

Chain K:

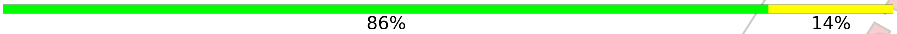

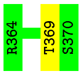

• Molecule 2: DAPK2 C-terminal peptide

Chain L:

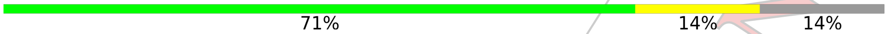

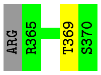

• Molecule 2: DAPK2 C-terminal peptide

Chain M:

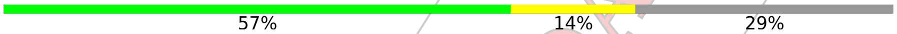

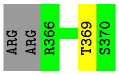

CONFIDENTIAL VALIDATION REPORT

4 Data and refinement statistics (i)

Property	Value	Source
Space group	H 3	Depositor
Cell constants<br>a, b, c, α, β, γ	205.50Å 205.50Å 74.27Å<br>90.00° 90.00° 120.00°	Depositor
Resolution (Å)	29.68 – 2.50<br>41.11 – 2.50	Depositor<br>EDS
% Data completeness<br>(in resolution range)	92.2 (29.68-2.50)<br>94.7 (41.11-2.50)	Depositor<br>EDS
R_{merge}	0.04	Depositor
R_{sym}	(Not available)	Depositor
$< I/\sigma(I) >$ [1]	2.28 (at 2.51Å)	Xtriage
Refinement program	PHENIX 1.16_3549	Depositor
R, R_{free}	0.223 , 0.259<br>0.223 , 0.259	Depositor<br>DCC
R_{free} test set	1889 reflections (4.93%)	wwPDB-VP
Wilson B-factor (Å^2)	44.9	Xtriage
Anisotropy	0.322	Xtriage
Bulk solvent k_{sol}(e/Å^3), B_{sol}(Å^2)	0.34 , 42.4	EDS
L-test for twinning[2]	$< \|L\| >$ = 0.51, $< L^2 >$ = 0.35	Xtriage
Estimated twinning fraction	0.001 for h,-h-k,-l	Xtriage
F_o,F_c correlation	0.93	EDS
Total number of atoms	7579	wwPDB-VP
Average B, all atoms (Å^2)	52.0	wwPDB-VP

Xtriage's analysis on translational NCS is as follows: *The largest off-origin peak in the Patterson function is 5.28% of the height of the origin peak. No significant pseudotranslation is detected.*

[1] Intensities estimated from amplitudes.

[2] Theoretical values of $< |L| >$, $< L^2 >$ for acentric reflections are 0.5, 0.333 respectively for untwinned datasets, and 0.375, 0.2 for perfectly twinned datasets.

CONFIDENTIAL VALIDATION REPORT

5 Model quality (i)

5.1 Standard geometry (i)

Bond lengths and bond angles in the following residue types are not validated in this section: TPO, FSC

The Z score for a bond length (or angle) is the number of standard deviations the observed value is removed from the expected value. A bond length (or angle) with $|Z| > 5$ is considered an outlier worth inspection. RMSZ is the root-mean-square of all Z scores of the bond lengths (or angles).

Mol	Chain	Bond lengths		Bond angles	
		RMSZ	#|Z| >5	RMSZ	#|Z| >5
1	A	0.24	0/1822	0.39	0/2460
1	B	0.24	0/1749	0.40	0/2364
1	C	0.25	0/1824	0.39	0/2462
1	D	0.25	0/1821	0.40	0/2465
2	J	0.19	0/33	0.35	0/39
2	K	0.19	0/32	0.37	0/39
2	L	0.19	0/27	0.31	0/32
2	M	0.16	0/28	0.29	0/32
All	All	0.25	0/7336	0.40	0/9893

There are no bond length outliers.

There are no bond angle outliers.

There are no chirality outliers.

There are no planarity outliers.

5.2 Too-close contacts (i)

In the following table, the Non-H and H(model) columns list the number of non-hydrogen atoms and hydrogen atoms in the chain respectively. The H(added) column lists the number of hydrogen atoms added and optimized by MolProbity. The Clashes column lists the number of clashes within the asymmetric unit, whereas Symm-Clashes lists symmetry-related clashes.

Mol	Chain	Non-H	H(model)	H(added)	Clashes	Symm-Clashes
1	A	1796	0	1760	16	0
1	B	1724	0	1673	9	0
1	C	1797	0	1753	10	0
1	D	1795	0	1722	16	0
2	J	46	0	34	1	0

Continued on next page...

CONFIDENTIAL VALIDATION REPORT

Continued from previous page...

Mol	Chain	Non-H	H(model)	H(added)	Clashes	Symm-Clashes
2	K	45	0	25	0	0
2	L	40	0	23	0	0
2	M	41	0	32	0	0
3	A	48	0	55	1	0
3	C	48	0	55	1	0
3	D	48	0	55	1	0
4	A	67	0	0	2	0
4	B	39	0	0	0	0
4	C	29	0	0	0	0
4	D	12	0	0	1	0
4	J	2	0	0	0	0
4	L	1	0	0	0	0
4	M	1	0	0	0	0
All	All	7579	0	7187	48	0

The all-atom clashscore is defined as the number of clashes found per 1000 atoms (including hydrogen atoms). The all-atom clashscore for this structure is 3.

All (48) close contacts within the same asymmetric unit are listed below, sorted by their clash magnitude.

Atom-1	Atom-2	Interatomic distance (Å)	Clash overlap (Å)
1:A:13:LEU:HD11	1:C:82:VAL:HG22	1.72	0.70
1:B:168:THR:HG21	1:B:207:GLU:HB2	1.76	0.67
1:C:165:MET:HE1	1:C:171:ILE:HB	1.79	0.64
1:A:82:VAL:HG22	1:C:13:LEU:HD11	1.82	0.62
1:A:192:GLN:NE2	4:A:403:HOH:O	2.34	0.61
1:B:113:SER:HB2	1:B:116:GLN:HG3	1.86	0.58
1:D:23:MET:HG2	1:D:48:ALA:HB2	1.87	0.56
1:D:195:HIS:O	1:D:199:THR:HG23	2.05	0.56
1:A:28:LYS:NZ	1:A:99:ASP:OD1	2.34	0.55
1:A:87:GLU:HA	1:A:90:GLU:HB2	1.88	0.55
1:A:77:LYS:HA	1:A:77:LYS:HE2	1.90	0.53
1:C:227:ARG:O	1:C:231:THR:HG23	2.10	0.52
1:B:28:LYS:NZ	1:B:99:ASP:OD1	2.35	0.51
1:A:218:ASP:OD1	1:A:218:ASP:N	2.44	0.51
1:C:70:THR:HG22	1:C:79:ILE:HG13	1.93	0.51
1:C:197:ALA:HB3	1:C:230:LEU:HD21	1.94	0.50
1:B:198:LYS:NZ	1:B:202:ASP:OD2	2.43	0.50
1:D:4:ARG:N	4:D:402:HOH:O	2.43	0.50
1:A:63:ILE:HG12	1:C:13:LEU:HD22	1.94	0.50
1:D:92:GLU:O	1:D:96:VAL:HG23	2.11	0.49

Continued on next page...

CONFIDENTIAL VALIDATION REPORT

Continued from previous page...

Atom-1	Atom-2	Interatomic distance (Å)	Clash overlap (Å)
1:D:165:MET:O	1:D:172:ARG:NH1	2.44	0.49
1:C:218:ASP:N	1:C:218:ASP:OD1	2.46	0.48
1:D:96:VAL:O	1:D:100:VAL:HG22	2.14	0.48
1:D:170:PRO:HB3	1:D:218:ASP:HB3	1.95	0.48
1:A:179:TYR:CD2	1:A:196:LEU:HD11	2.48	0.48
1:A:113:SER:H	1:A:116:GLN:HG3	1.78	0.47
1:B:83:ARG:O	1:B:87:GLU:HG3	2.15	0.45
1:D:118:GLU:HG2	1:D:171:ILE:HD12	1.98	0.45
1:A:83:ARG:O	1:A:87:GLU:HG2	2.16	0.45
1:D:113:SER:OG	1:D:114:GLU:N	2.49	0.45
1:D:202:ASP:HA	1:D:205:ILE:HG12	1.99	0.45
1:A:50:LYS:NZ	4:A:409:HOH:O	2.49	0.45
1:B:179:TYR:HB3	1:B:196:LEU:HD21	2.00	0.43
1:D:8:VAL:O	1:D:12:ARG:HG3	2.19	0.43
1:A:67:GLU:OE1	1:A:86:ARG:NH2	2.51	0.43
1:C:125:LYS:HE3	3:C:301:FSC:H262	2.00	0.43
1:A:110:LYS:O	1:D:110:LYS:NZ	2.38	0.43
1:D:145:THR:O	1:D:148:GLU:HB3	2.18	0.42
1:D:218:ASP:O	1:D:222:ILE:HG13	2.19	0.42
3:D:301:FSC:H28	3:D:301:FSC:H172	2.00	0.42
1:B:190:PRO:HB2	2:J:365:ARG:NH2	2.34	0.42
1:B:114:GLU:HA	1:B:120:LYS:HE3	2.02	0.41
1:A:125:LYS:HE3	3:A:301:FSC:H262	2.02	0.41
1:B:232:LEU:HD22	1:B:233:TRP:HD1	1.84	0.41
1:C:129:ASP:OD1	1:C:154:TYR:OH	2.28	0.41
1:D:155:SER:O	1:D:159:GLU:HG2	2.20	0.41
1:A:152:LYS:HE3	1:A:152:LYS:HB3	1.75	0.41
1:D:135:ALA:HB2	1:D:146:VAL:HG23	2.04	0.40

There are no symmetry-related clashes.

5.3 Torsion angles (i)

5.3.1 Protein backbone (i)

In the following table, the Percentiles column shows the percent Ramachandran outliers of the chain as a percentile score with respect to all X-ray entries followed by that with respect to entries of similar resolution.

The Analysed column shows the number of residues for which the backbone conformation was analysed, and the total number of residues.

CONFIDENTIAL VALIDATION REPORT

Mol	Chain	Analysed	Favoured	Allowed	Outliers	Percentiles	
1	A	220/236 (93%)	218 (99%)	2 (1%)	0	100	100
1	B	211/236 (89%)	209 (99%)	2 (1%)	0	100	100
1	C	221/236 (94%)	221 (100%)	0	0	100	100
1	D	225/236 (95%)	224 (100%)	1 (0%)	0	100	100
2	J	3/7 (43%)	3 (100%)	0	0	100	100
2	K	4/7 (57%)	4 (100%)	0	0	100	100
2	L	3/7 (43%)	3 (100%)	0	0	100	100
2	M	2/7 (29%)	2 (100%)	0	0	100	100
All	All	889/972 (92%)	884 (99%)	5 (1%)	0	100	100

There are no Ramachandran outliers to report.

5.3.2 Protein sidechains (i)

In the following table, the Percentiles column shows the percent sidechain outliers of the chain as a percentile score with respect to all X-ray entries followed by that with respect to entries of similar resolution.

The Analysed column shows the number of residues for which the sidechain conformation was analysed, and the total number of residues.

Mol	Chain	Analysed	Rotameric	Outliers	Percentiles	
1	A	189/207 (91%)	186 (98%)	3 (2%)	62	84
1	B	182/207 (88%)	180 (99%)	2 (1%)	73	89
1	C	190/207 (92%)	185 (97%)	5 (3%)	46	72
1	D	187/207 (90%)	182 (97%)	5 (3%)	44	71
2	J	4/6 (67%)	3 (75%)	1 (25%)	0	1
2	K	3/6 (50%)	3 (100%)	0	100	100
2	L	3/6 (50%)	3 (100%)	0	100	100
2	M	4/6 (67%)	4 (100%)	0	100	100
All	All	762/852 (89%)	746 (98%)	16 (2%)	53	78

All (16) residues with a non-rotameric sidechain are listed below:

Mol	Chain	Res	Type
1	A	64	SER
1	A	86	ARG

Continued on next page...

CONFIDENTIAL VALIDATION REPORT

Continued from previous page...

Mol	Chain	Res	Type
1	A	179	TYR
1	B	161	SER
1	B	179	TYR
1	C	65	SER
1	C	161	SER
1	C	162	LYS
1	C	179	TYR
1	C	228	ASP
1	D	149	SER
1	D	150	SER
1	D	155	SER
1	D	192	GLN
1	D	215	SER
2	J	370	SER

Sometimes sidechains can be flipped to improve hydrogen bonding and reduce clashes. All (1) such sidechains are listed below:

Mol	Chain	Res	Type
1	B	6	GLN

5.3.3 RNA (i)

There are no RNA molecules in this entry.

5.4 Non-standard residues in protein, DNA, RNA chains (i)

4 non-standard protein/DNA/RNA residues are modelled in this entry.

In the following table, the Counts columns list the number of bonds (or angles) for which Mogul statistics could be retrieved, the number of bonds (or angles) that are observed in the model and the number of bonds (or angles) that are defined in the Chemical Component Dictionary. The Link column lists molecule types, if any, to which the group is linked. The Z score for a bond length (or angle) is the number of standard deviations the observed value is removed from the expected value. A bond length (or angle) with $|Z| > 2$ is considered an outlier worth inspection. RMSZ is the root-mean-square of all Z scores of the bond lengths (or angles).

Mol	Type	Chain	Res	Link	Bond lengths			Bond angles		
					Counts	RMSZ	$\#\|Z\| > 2$	Counts	RMSZ	$\#\|Z\| > 2$
2	TPO	L	369	2	8,10,11	1.11	0	10,14,16	1.72	2 (20%)
2	TPO	K	369	2	8,10,11	1.60	1 (12%)	10,14,16	1.71	1 (10%)
2	TPO	J	369	2	8,10,11	1.58	1 (12%)	10,14,16	1.68	2 (20%)

CONFIDENTIAL VALIDATION REPORT

Mol	Type	Chain	Res	Link	Bond lengths			Bond angles		
					Counts	RMSZ	#|Z| > 2	Counts	RMSZ	#|Z| > 2
2	TPO	M	369	2	8,10,11	1.57	1 (12%)	10,14,16	1.78	1 (10%)

In the following table, the Chirals column lists the number of chiral outliers, the number of chiral centers analysed, the number of these observed in the model and the number defined in the Chemical Component Dictionary. Similar counts are reported in the Torsion and Rings columns. '-' means no outliers of that kind were identified.

Mol	Type	Chain	Res	Link	Chirals	Torsions	Rings
2	TPO	L	369	2	-	2/9/11/13	-
2	TPO	K	369	2	-	1/9/11/13	-
2	TPO	J	369	2	-	2/9/11/13	-
2	TPO	M	369	2	-	2/9/11/13	-

All (3) bond length outliers are listed below:

Mol	Chain	Res	Type	Atoms	Z	Observed(Å)	Ideal(Å)
2	K	369	TPO	P-O1P	3.39	1.61	1.50
2	M	369	TPO	P-O1P	3.37	1.61	1.50
2	J	369	TPO	P-O1P	3.32	1.61	1.50

All (6) bond angle outliers are listed below:

Mol	Chain	Res	Type	Atoms	Z	Observed(^{o})	Ideal(^{o})
2	M	369	TPO	P-OG1-CB	-4.94	108.30	123.21
2	K	369	TPO	P-OG1-CB	-4.75	108.87	123.21
2	L	369	TPO	P-OG1-CB	-4.38	109.97	123.21
2	J	369	TPO	P-OG1-CB	-4.27	110.31	123.21
2	L	369	TPO	CG2-CB-CA	-2.26	108.70	113.16
2	J	369	TPO	CG2-CB-CA	-2.11	108.99	113.16

There are no chirality outliers.

All (7) torsion outliers are listed below:

Mol	Chain	Res	Type	Atoms
2	J	369	TPO	CB-OG1-P-O1P
2	L	369	TPO	CB-OG1-P-O2P
2	M	369	TPO	CB-OG1-P-O1P
2	J	369	TPO	O-C-CA-CB
2	K	369	TPO	O-C-CA-CB
2	L	369	TPO	O-C-CA-CB

Continued on next page...

CONFIDENTIAL VALIDATION REPORT

Continued from previous page...

Mol	Chain	Res	Type	Atoms
2	M	369	TPO	O-C-CA-CB

There are no ring outliers.

No monomer is involved in short contacts.

5.5 Carbohydrates (i)

There are no monosaccharides in this entry.

5.6 Ligand geometry (i)

3 ligands are modelled in this entry.

In the following table, the Counts columns list the number of bonds (or angles) for which Mogul statistics could be retrieved, the number of bonds (or angles) that are observed in the model and the number of bonds (or angles) that are defined in the Chemical Component Dictionary. The Link column lists molecule types, if any, to which the group is linked. The Z score for a bond length (or angle) is the number of standard deviations the observed value is removed from the expected value. A bond length (or angle) with $|Z| > 2$ is considered an outlier worth inspection. RMSZ is the root-mean-square of all Z scores of the bond lengths (or angles).

Mol	Type	Chain	Res	Link	Bond lengths			Bond angles		
					Counts	RMSZ	$\#\|Z\| > 2$	Counts	RMSZ	$\#\|Z\| > 2$
3	FSC	C	301	-	46,51,51	1.17	6 (13%)	47,77,77	1.98	9 (19%)
3	FSC	A	301	-	46,51,51	1.23	4 (8%)	47,77,77	1.89	7 (14%)
3	FSC	D	301	-	46,51,51	1.33	5 (10%)	47,77,77	2.11	11 (23%)

In the following table, the Chirals column lists the number of chiral outliers, the number of chiral centers analysed, the number of these observed in the model and the number defined in the Chemical Component Dictionary. Similar counts are reported in the Torsion and Rings columns. '-' means no outliers of that kind were identified.

Mol	Type	Chain	Res	Link	Chirals	Torsions	Rings
3	FSC	C	301	-	-	4/29/103/103	0/4/4/4
3	FSC	A	301	-	-	2/29/103/103	0/4/4/4
3	FSC	D	301	-	-	14/29/103/103	0/4/4/4

All (15) bond length outliers are listed below:

CONFIDENTIAL VALIDATION REPORT

Mol	Chain	Res	Type	Atoms	Z	Observed(Å)	Ideal(Å)
3	D	301	FSC	C19-C15	-4.81	1.52	1.56
3	A	301	FSC	C19-C15	-4.64	1.52	1.56
3	D	301	FSC	C33-C30	4.30	1.62	1.53
3	C	301	FSC	C19-C15	-3.47	1.53	1.56
3	C	301	FSC	O34-C28	-2.64	1.40	1.44
3	C	301	FSC	C15-C9	2.50	1.55	1.53
3	A	301	FSC	C33-C30	2.44	1.58	1.53
3	C	301	FSC	C10-C6	2.34	1.55	1.52
3	D	301	FSC	O13-C6	-2.25	1.39	1.43
3	C	301	FSC	O13-C6	-2.23	1.39	1.43
3	C	301	FSC	C33-C30	2.17	1.57	1.53
3	A	301	FSC	O34-C28	-2.14	1.41	1.44
3	D	301	FSC	C10-C6	2.09	1.55	1.52
3	A	301	FSC	O13-C6	-2.09	1.40	1.43
3	D	301	FSC	O34-C28	-2.01	1.41	1.44

All (27) bond angle outliers are listed below:

Mol	Chain	Res	Type	Atoms	Z	Observed(^{o})	Ideal(^{o})
3	C	301	FSC	C15-C19-C12	6.94	127.00	118.11
3	A	301	FSC	C15-C19-C12	6.51	126.44	118.11
3	D	301	FSC	C15-C19-C12	6.40	126.31	118.11
3	A	301	FSC	C7-C2-C6	-5.61	104.56	112.81
3	D	301	FSC	C7-C2-C6	-5.48	104.76	112.81
3	C	301	FSC	C7-C2-C6	-5.47	104.77	112.81
3	D	301	FSC	C35-C30-C33	-4.73	102.22	112.09
3	D	301	FSC	O22-C30-C33	4.29	117.48	109.69
3	C	301	FSC	C19-C15-C9	4.22	116.83	112.39
3	A	301	FSC	C19-C12-C5	3.97	135.04	130.72
3	D	301	FSC	C19-C12-C5	3.75	134.81	130.72
3	D	301	FSC	C19-C15-C9	3.69	116.27	112.39
3	C	301	FSC	C19-C12-C5	3.68	134.72	130.72
3	A	301	FSC	C19-C15-C9	3.50	116.07	112.39
3	C	301	FSC	C9-C3-C1	-3.46	108.00	112.98
3	C	301	FSC	C35-C30-C33	-3.35	105.10	112.09
3	D	301	FSC	C9-C3-C1	-3.16	108.43	112.98
3	A	301	FSC	C9-C3-C1	-2.97	108.70	112.98
3	D	301	FSC	C14-O22-C30	2.87	119.33	113.69
3	A	301	FSC	O34-C40-C42	2.47	115.63	111.09
3	D	301	FSC	O34-C40-C42	2.46	115.61	111.09
3	A	301	FSC	C35-C30-C33	-2.46	106.96	112.09
3	C	301	FSC	O34-C40-C42	2.33	115.37	111.09
3	D	301	FSC	C18-C11-C4	-2.11	108.02	111.31

Continued on next page...

CONFIDENTIAL VALIDATION REPORT

Continued from previous page...

Mol	Chain	Res	Type	Atoms	Z	Observed(°)	Ideal(°)
3	C	301	FSC	C21-C28-C33	-2.07	107.87	110.85
3	C	301	FSC	C18-C11-C4	-2.04	108.13	111.31
3	D	301	FSC	C28-C33-C30	2.03	113.99	109.66

There are no chirality outliers.

All (20) torsion outliers are listed below:

Mol	Chain	Res	Type	Atoms
3	A	301	FSC	C33-C30-C35-O41
3	D	301	FSC	C4-C11-C17-O24
3	D	301	FSC	C18-C11-C17-O24
3	D	301	FSC	O41-C44-C45-C48
3	D	301	FSC	C42-C40-O34-C28
3	D	301	FSC	O43-C40-O34-C28
3	A	301	FSC	O22-C30-C35-O41
3	C	301	FSC	C33-C30-C35-O41
3	D	301	FSC	C36-C31-O24-C17
3	D	301	FSC	O22-C30-C35-O41
3	D	301	FSC	C33-C30-C35-O41
3	D	301	FSC	O37-C31-O24-C17
3	D	301	FSC	C20-C27-O32-C38
3	C	301	FSC	O22-C30-C35-O41
3	C	301	FSC	C18-C11-C17-O24
3	C	301	FSC	C20-C27-O32-C38
3	D	301	FSC	C21-C28-O34-C40
3	D	301	FSC	C33-C28-O34-C40
3	D	301	FSC	C47-C44-C45-C48
3	D	301	FSC	C46-C44-C45-C48

There are no ring outliers.

3 monomers are involved in 3 short contacts:

Mol	Chain	Res	Type	Clashes	Symm-Clashes
3	C	301	FSC	1	0
3	A	301	FSC	1	0
3	D	301	FSC	1	0

The following is a two-dimensional graphical depiction of Mogul quality analysis of bond lengths, bond angles, torsion angles, and ring geometry for all instances of the Ligand of Interest. In addition, ligands with molecular weight > 250 and outliers as shown on the validation Tables will also be included. For torsion angles, if less then 5% of the Mogul distribution of torsion angles is within 10 degrees of the torsion angle in question, then that torsion angle is considered an outlier.

CONFIDENTIAL VALIDATION REPORT

Any bond that is central to one or more torsion angles identified as an outlier by Mogul will be highlighted in the graph. For rings, the root-mean-square deviation (RMSD) between the ring in question and similar rings identified by Mogul is calculated over all ring torsion angles. If the average RMSD is greater than 60 degrees and the minimal RMSD between the ring in question and any Mogul-identified rings is also greater than 60 degrees, then that ring is considered an outlier. The outliers are highlighted in purple. The color gray indicates Mogul did not find sufficient equivalents in the CSD to analyse the geometry.

Ligand FSC C 301

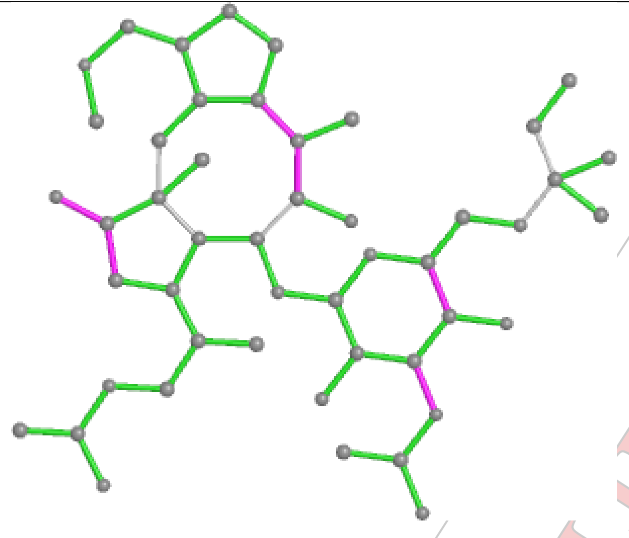

Bond lengths

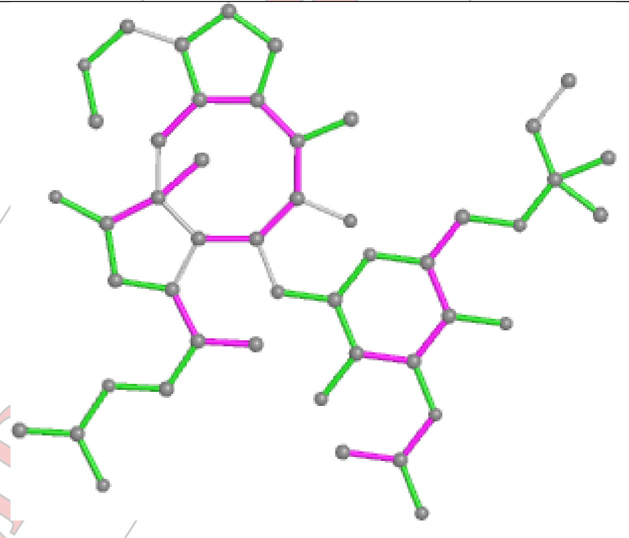

Bond angles

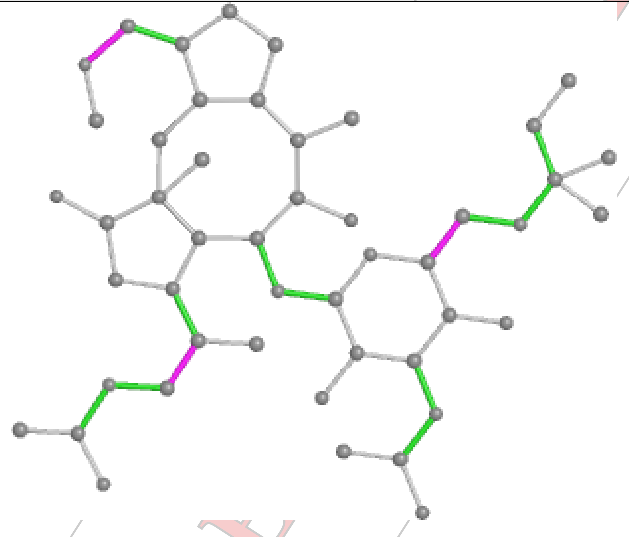

Torsions

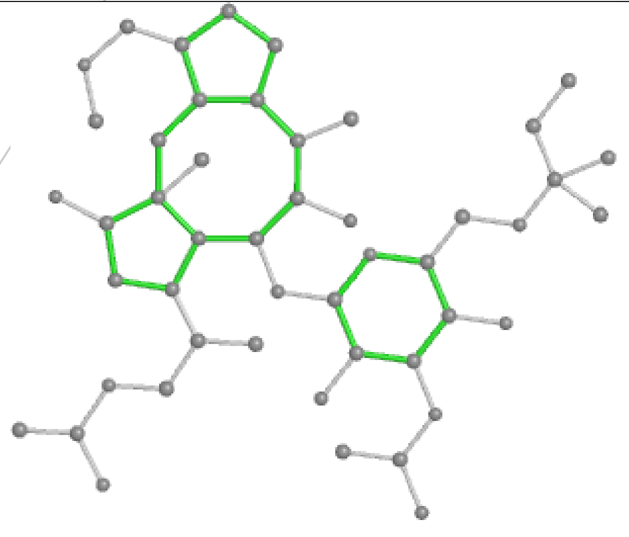

Rings

CONFIDENTIAL REPORT

Ligand FSC A 301

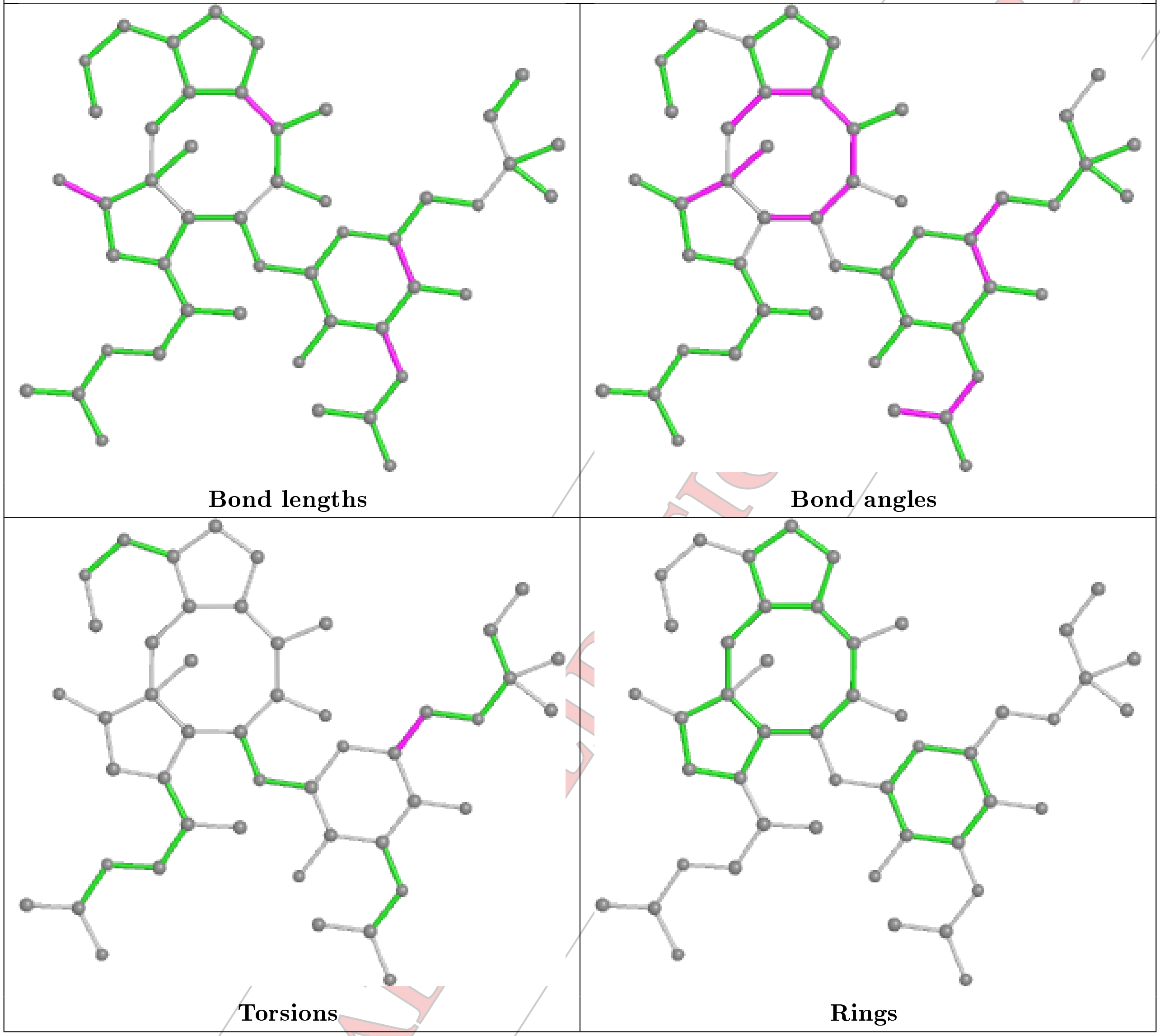

CONFIDENTIAL

Ligand FSC D 301

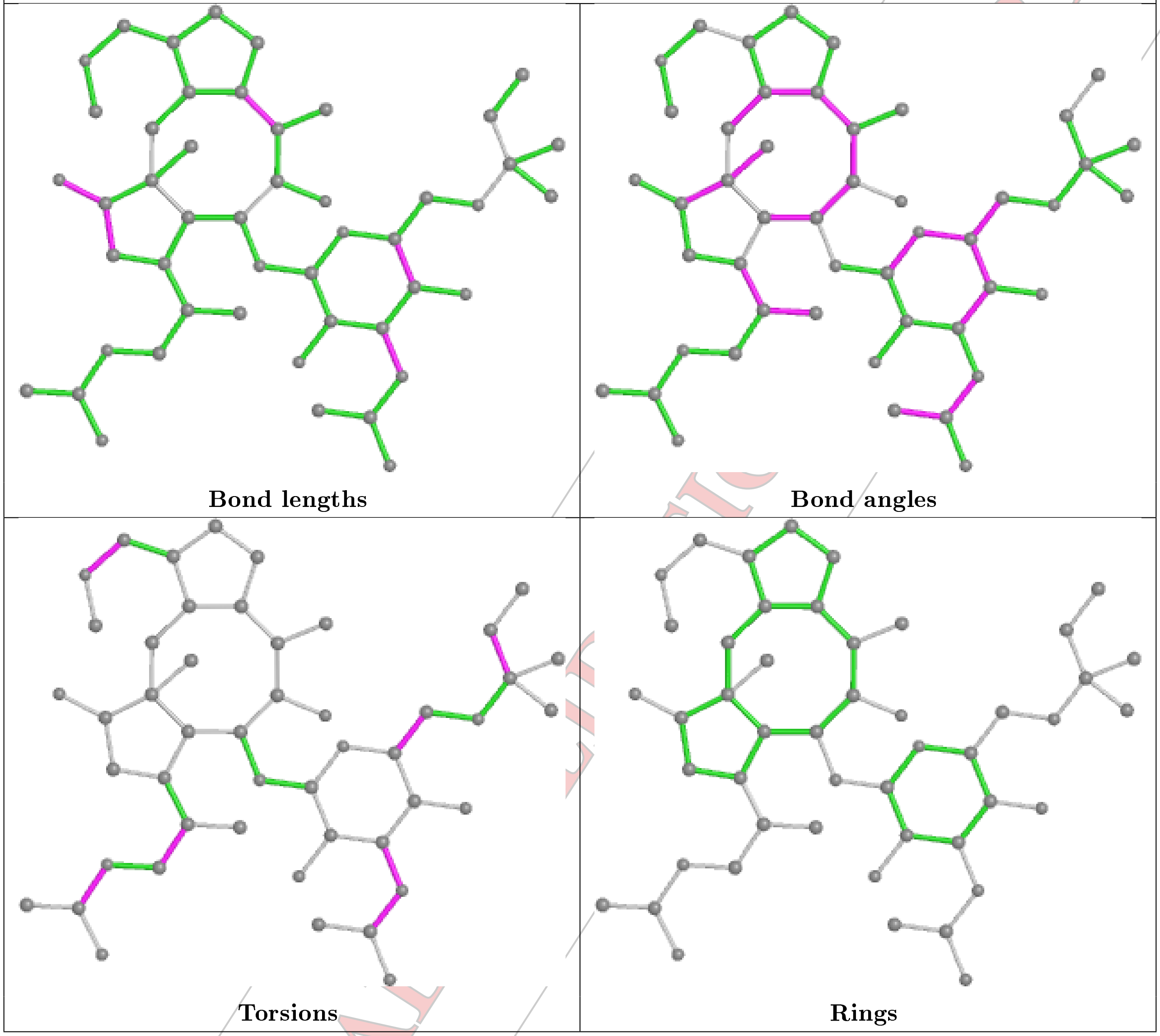

5.7 Other polymers (i)

There are no such residues in this entry.

5.8 Polymer linkage issues (i)

There are no chain breaks in this entry.

CONFIDENTIAL

6 Fit of model and data (i)

6.1 Protein, DNA and RNA chains (i)

In the following table, the column labelled '#RSRZ> 2' contains the number (and percentage) of RSRZ outliers, followed by percent RSRZ outliers for the chain as percentile scores relative to all X-ray entries and entries of similar resolution. The OWAB column contains the minimum, median, 95th percentile and maximum values of the occupancy-weighted average B-factor per residue. The column labelled 'Q< 0.9' lists the number of (and percentage) of residues with an average occupancy less than 0.9.

Mol	Chain	Analysed	<RSRZ>	#RSRZ>2			OWAB(Å^2)	Q<0.9
1	A	226/236 (95%)	-0.01	0	100	100	32, 44, 60, 70	0
1	B	217/236 (91%)	0.05	2 (0%)	84	86	32, 46, 69, 81	0
1	C	225/236 (95%)	0.28	4 (1%)	68	71	34, 52, 76, 89	0
1	D	229/236 (97%)	0.30	3 (1%)	77	79	39, 59, 77, 101	0
2	J	5/7 (71%)	0.09	0	100	100	39, 46, 51, 67	0
2	K	6/7 (85%)	-0.41	0	100	100	49, 61, 77, 79	0
2	L	5/7 (71%)	0.33	0	100	100	56, 63, 67, 74	0
2	M	4/7 (57%)	0.79	0	100	100	55, 56, 64, 71	0
All	All	917/972 (94%)	0.16	9 (0%)	82	84	32, 51, 74, 101	0

All (9) RSRZ outliers are listed below:

Mol	Chain	Res	Type	RSRZ
1	D	8	VAL	3.7
1	B	79	ILE	2.9
1	D	233	TRP	2.7
1	D	9	GLN	2.6
1	C	191	GLU	2.4
1	C	208	LEU	2.4
1	C	190	PRO	2.2
1	B	208	LEU	2.1
1	C	141	GLU	2.1

6.2 Non-standard residues in protein, DNA, RNA chains (i)

In the following table, the Atoms column lists the number of modelled atoms in the group and the number defined in the chemical component dictionary. The B-factors column lists the minimum, median, 95th percentile and maximum values of B factors of atoms in the group. The column

CONFIDENTIAL VALIDATION REPORT

labelled 'Q< 0.9' lists the number of atoms with occupancy less than 0.9.

Mol	Type	Chain	Res	Atoms	RSCC	RSR	B-factors($Å^2$)	Q<0.9
2	TPO	M	369	11/12	0.96	0.17	40,51,57,59	0
2	TPO	L	369	11/12	0.97	0.17	35,46,58,59	0
2	TPO	K	369	11/12	0.97	0.16	33,40,46,50	0
2	TPO	J	369	11/12	0.99	0.14	27,32,40,45	0

6.3 Carbohydrates ⓘ

There are no monosaccharides in this entry.

6.4 Ligands ⓘ

In the following table, the Atoms column lists the number of modelled atoms in the group and the number defined in the chemical component dictionary. The B-factors column lists the minimum, median, 95th percentile and maximum values of B factors of atoms in the group. The column labelled 'Q< 0.9' lists the number of atoms with occupancy less than 0.9.

Mol	Type	Chain	Res	Atoms	RSCC	RSR	B-factors($Å^2$)	Q<0.9
3	FSC	D	301	48/48	0.86	0.22	44,59,76,85	0
3	FSC	C	301	48/48	0.88	0.18	43,70,78,80	0
3	FSC	A	301	48/48	0.93	0.17	29,44,56,62	0

The following is a graphical depiction of the model fit to experimental electron density of all instances of the Ligand of Interest. In addition, ligands with molecular weight > 250 and outliers as shown on the geometry validation Tables will also be included. Each fit is shown from different orientation to approximate a three-dimensional view.

CONFIDENTIAL VALIDATION REPORT

Electron density around FSC D 301:

$2mF_o$-DF_c (at 0.7 rmsd) in gray
mF_o-DF_c (at 3 rmsd) in purple (negative)
and green (positive)

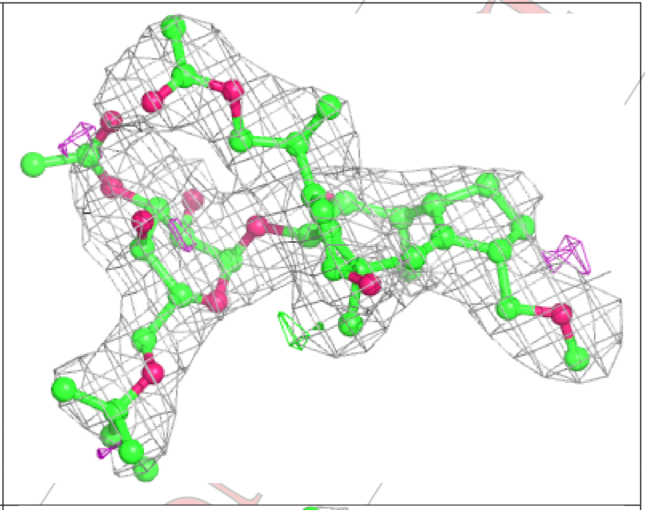

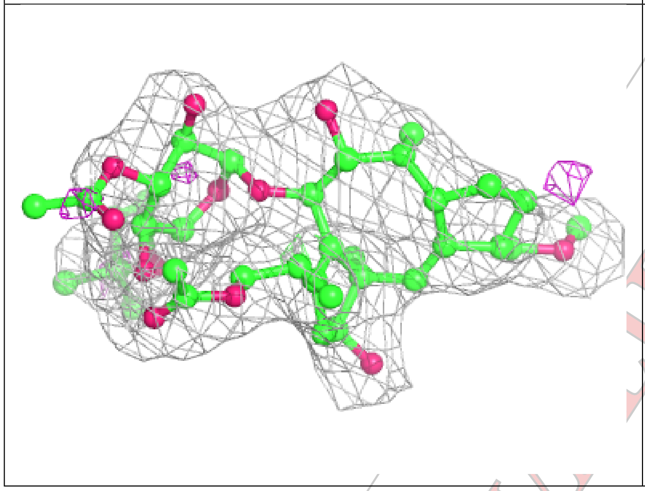

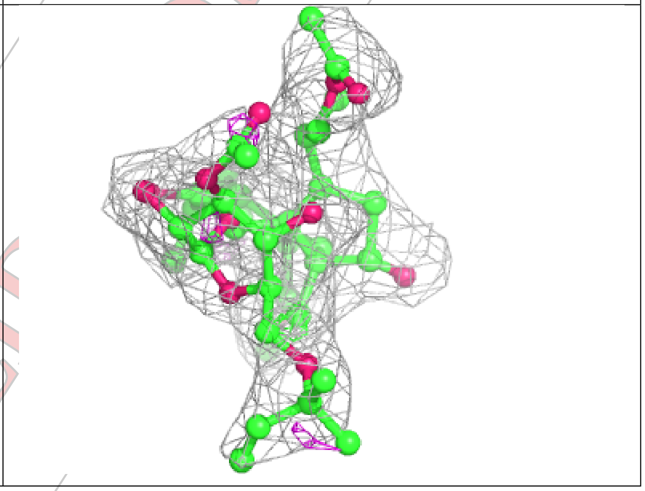

CONFIDENTIAL

Electron density around FSC C 301:

2mF$_o$-DF$_c$ (at 0.7 rmsd) in gray
mF$_o$-DF$_c$ (at 3 rmsd) in purple (negative)
and green (positive)

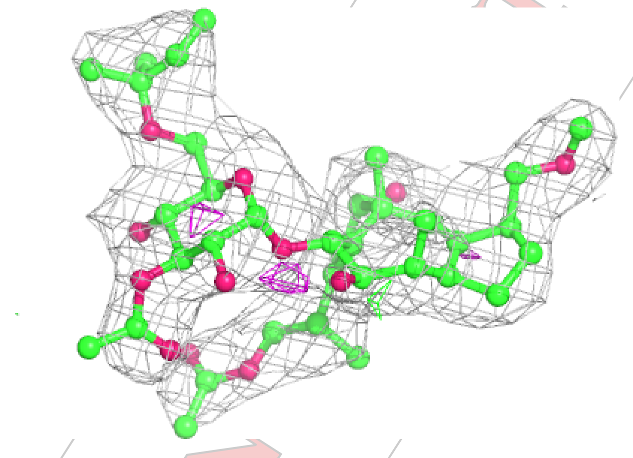

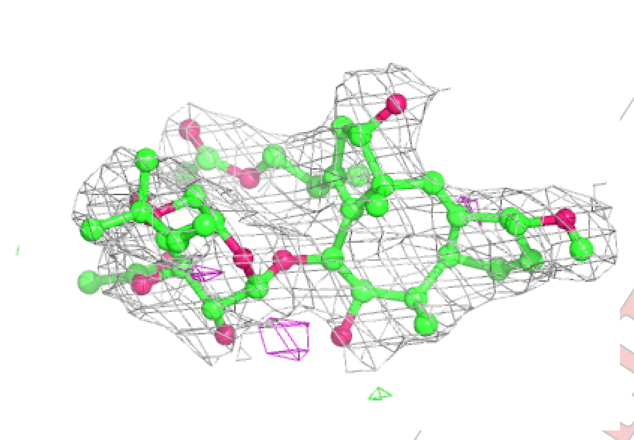

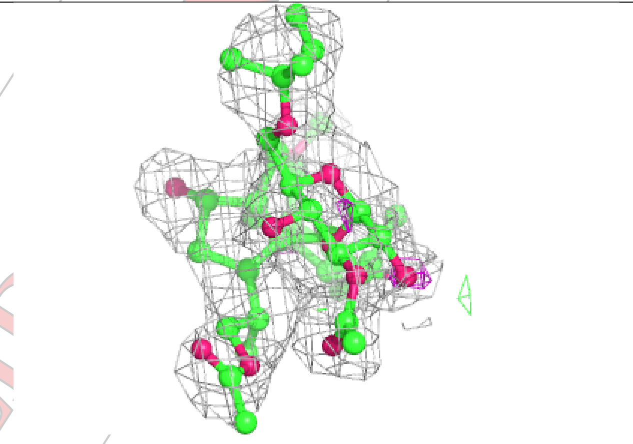

CONFIDENTIAL

Electron density around FSC A 301:

$2mF_o$-DF_c (at 0.7 rmsd) in gray
mF_o-DF_c (at 3 rmsd) in purple (negative)
and green (positive)

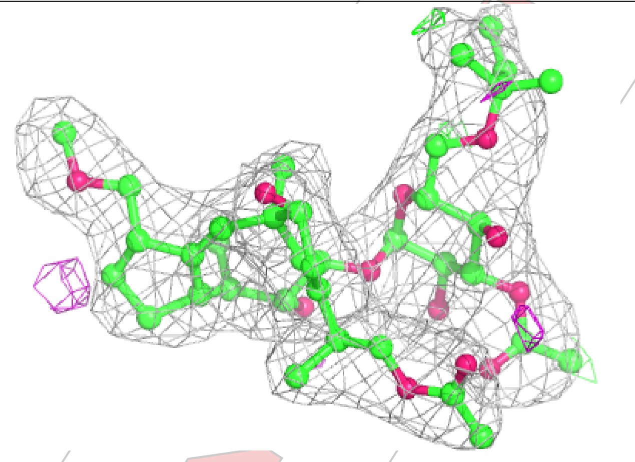

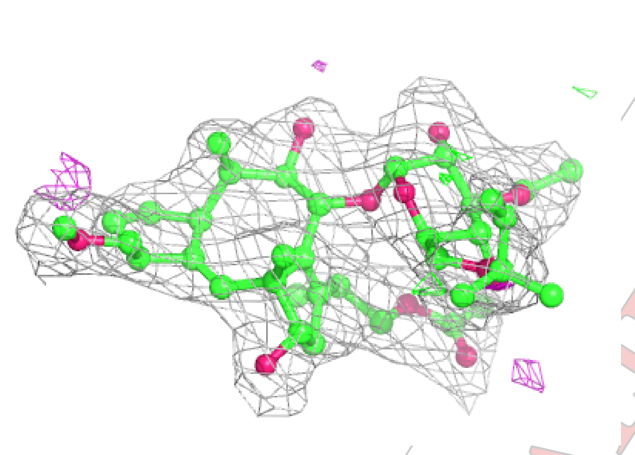

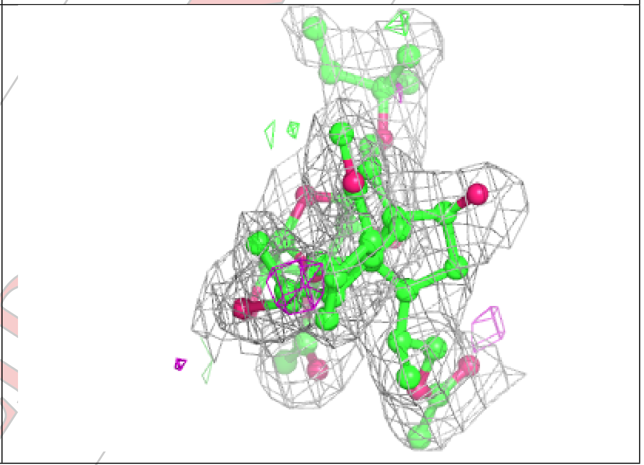

6.5 Other polymers (i)

There are no such residues in this entry.

CONFIDENTIAL VALIDATION
